# Shared genetic links between hypothyroidism and psychiatric disorders: evidence from a comprehensive genetic analysis

**DOI:** 10.3389/fendo.2024.1370019

**Published:** 2024-06-06

**Authors:** Jianlong Zhou, Lv Zhu

**Affiliations:** ^1^ People’s Hospital of Deyang City, Affiliated to Chengdu University of Traditional Chinese Medicine, Deyang, China; ^2^ Department of Integrative Medicine, West China Hospital, Sichuan University, Chengdu, China

**Keywords:** hypothyroidism, psychiatric disorders, genome-wide association study, shared genetic, Mendelian randomization

## Abstract

**Background:**

Epidemiologic studies have suggested co-morbidity between hypothyroidism and psychiatric disorders. However, the shared genetic etiology and causal relationship between them remain currently unclear.

**Methods:**

We assessed the genetic correlations between hypothyroidism and psychiatric disorders [anxiety disorders (ANX), schizophrenia (SCZ), major depressive disorder (MDD), and bipolar disorder (BIP)] using summary association statistics from genome-wide association studies (GWAS). Two disease-associated pleiotropic risk loci and genes were identified, and pathway enrichment, tissue enrichment, and other analyses were performed to determine their specific functions. Furthermore, we explored the causal relationship between them through Mendelian randomization (MR) analysis.

**Results:**

We found significant genetic correlations between hypothyroidism with ANX, SCZ, and MDD, both in the Linkage disequilibrium score regression (LDSC) approach and the high-definition likelihood (HDL) approach. Meanwhile, the strongest correlation was observed between hypothyroidism and MDD (LDSC: rg=0.264, *P*=7.35×10^-12^; HDL: rg=0.304, *P*=4.14×10^-17^). We also determined a significant genetic correlation between MDD with free thyroxine (FT4) and thyroid-stimulating hormone (TSH) levels. A total of 30 pleiotropic risk loci were identified between hypothyroidism and psychiatric disorders, of which the 15q14 locus was identified in both ANX and SCZ (*P* values are 6.59×10^-11^ and 2.10×10^-12^, respectively) and the 6p22.1 locus was identified in both MDD and SCZ (*P* values are 1.05×10^-8^ and 5.75×10^-14^, respectively). Sixteen pleiotropic risk loci were identified between MDD and indicators of thyroid function, of which, four loci associated with MDD (1p32.3, 6p22.1, 10q21.1, 11q13.4) were identified in both FT4 normal level and Hypothyroidism. Further, 79 pleiotropic genes were identified using Magma gene analysis (*P*<0.05/18776 = 2.66×10^-6^). Tissue-specific enrichment analysis revealed that these genes were highly enriched into six brain-related tissues. The pathway analysis mainly involved nucleosome assembly and lipoprotein particles. Finally, our two-sample MR analysis showed a significant causal effect of MDD on the increased risk of hypothyroidism, and BIP may reduce TSH normal levels.

**Conclusions:**

Our findings not only provided evidence of a shared genetic etiology between hypothyroidism and psychiatric disorders, but also provided insights into the causal relationships and biological mechanisms that underlie their relationship. These findings contribute to a better understanding of the pleiotropy between hypothyroidism and psychiatric disorders, while having important implications for intervention and treatment goals for these disorders.

## 1 Introduction

Hypothyroidism is a common endocrine disorder caused by a decrease in the synthesis and secretion of thyroid hormone (TH) or a deficiency in the physiologic effect of TH, mainly including subclinical hypothyroidism (SCH) and overt hypothyroidism (OH) (1, 2). According to recent estimates, the overall prevalence of hypothyroidism was 4.70% in Europe (3) and 13.95% in the general population of China (4). It was defined as elevated thyroid-stimulating hormone (TSH) with low or normal free thyroxine (FT4) concentration (5). A study of a large UK twin cohort found that the heritability of TSH and FT4 was 65% and 39–80%, respectively (6). About 49% of patients with hypothyroidism have an autoimmune-related etiology (7). Hashimoto thyroiditis (HT), a chronic autoimmune thyroid disease, is the main cause of hypothyroidism (8) and has a strong genetic background (9). These suggested that genetic factors play an important role in the development of hypothyroidism. Psychiatric disorders are a wide range of mental health disorders that affect human emotions, thinking, and behavior (10). Psychiatric disorders have been recognized as a heavy burden on individual health care and the current healthcare system (11). Statistically, the global burden of psychiatric disorders accounts for 32.4% of years lived with disability (YLDs) and 13.0% of disability-adjusted life years (DALYs) (12). There are approximately 8 million deaths each year worldwide due to psychiatric disorders, which accounts for 14.3% of the deaths worldwide (13). Research has confirmed that schizophrenia (SCZ), major depressive disorder (MDD), and bipolar disorder (BIP) are major psychiatric disorders (MPDs) with high heritability (14). Anxiety disorders (ANX), a common psychiatric disorder, are also affected by genetic factors (15).

There is growing evidence of co-morbidity between hypothyroidism and psychiatric disorders (16, 17), and that thyroid function and psychiatric disorders may interact in both directions (18–20). For example, a population-based cross-sectional study found a high rate of hypothyroidism in patients with SCZ (21). A nested case-control study based on the Finnish Prenatal Study of Schizophrenia demonstrated an association between maternal hypothyroxinemia and increased odds of SCZ (22). Epidemiologic investigations have shown that in the hypothyroid phase of HT, 50% of patients were often accompanied by a depressive state, and hypothyroid patients were 3.3 times more likely to exhibit depressive symptoms than healthy controls (23). A study conducted in Alameda County found that maternal hypothyroxinemia was significantly associated with a higher risk of BIP in offspring (24). A clinical cross-sectional study demonstrated a higher prevalence of subclinical hypothyroidism in adolescents with depression compared to mentally healthy volunteers (25). Although several observational studies have shown hypothyroidism to be associated with ANX and depression (26, 27), contrary findings still exist. For instance, a large population-based study failed to find a link between hypothyroidism and anxiety or depression (28). These interesting findings prompted us to include ANX in the present study. These findings also suggest that hypothyroidism may have some shared genetic risk with psychiatric disorders, but the extent of genetic overlap is uncertain. Moreover, observational studies may be affected by confounding factors and reverse causation, so the causation between hypothyroidism and psychiatric disorders also remains unclear.

Notably, how to treat patients with both hypothyroidism and psychiatric disorders is an important issue for clinicians. TH replacement therapy is the primary treatment for OH (29). In recent years, this therapy has also been recognized as an effective option for adjunctive treatment of drug-resistant depression (30). However, research has found that antipsychotic medications may affect thyroid function in patients with psychiatric disorders. Quetiapine treatment in the acute phase of SCZ was strongly associated with the risk of new-onset hypothyroidism (31). Other studies have shown (32) that phenothiazines may increase thyrotropin-releasing hormone (TRH), further raising TSH levels. Tricyclic antidepressants and carbamazepine may decrease serum TH levels (32). Conversely, clozapine may reduce TRH and further decrease TSH levels (33). Lithium therapy is the first line of long-term treatment for BIP and may also lead to hypothyroidism (34). Therefore, it is necessary to conduct in-depth analyses for exploring the genetic etiology and causal relationship between hypothyroidism and psychiatric disorders. Further studies are needed to clarify whether there are common pleiotropic genetic variants and whether specific molecular biological pathways are involved between the two. This may be beneficial in addressing these important and common clinical issues.

In this study, we used pooled data from a large-scale genome-wide association study (GWAS) to investigate the genetic correlations and potential causal relationships between hypothyroidism and SCZ, MDD, BIP, and ANX. We assessed the genetic overlap between them using genetic correlation methods. Then, the corresponding pleiotropic loci and genes were identified using the pleiotropic analysis under composite null hypothesis (PLACO) method, and the genes were functionally annotated and tissue enriched. Finally, we implemented a bidirectional two-sample bidirectional two-sample Mendelian randomization (MR) analysis to assess the causal relationship between them.

## 2 Methods

### 2.1 GWAS summary statistics

#### 2.1.1 GWAS dataset for ANX, BIP, SCZ, and MDD

We analyzed GWAS summary datasets obtained from the Psychiatric Genomics Consortium (PGC) (https://www.med.unc.edu/pgc/download-results/) for four psychiatric disorders, including ANX, BIP, SCZ, and MDD. See **Supplementary Table S1** for data sources and basic information. The GWAS data from ANX incorporated genome-wide genotype data from 2,248 clinically well characterized Parkinson’s disease (PD) patients and 7,992 ethnically matched controls. It was also validated in an independent sample of 2,408 PD patients and 228,470 controls from Denmark, Iceland, and the Netherlands. Data for BIP came from a GWAS study that included 20,352 cases and 31,358 controls of European ancestry, which was validated in an additional 9,412 cases and 137,760 controls. The study identified 19 risk loci associated with BIP and found a strong genetic correlation between BIP with SCZ and MDD. Data for MDD came from a GWAS meta-analysis of 135,458 cases and 344,901 controls identified 44 independent and significant loci. The genetic findings were associated with clinical features of MDD and relevant brain regions exhibiting anatomical differences in cases. To ensure no sample overlap with the thyroid data, we selected a dataset that did not contain the UK Biobank (UKBB). Data for SCZ came from a two-stage GWAS study of up to 76,755 SCZ patients and 243,649 control individuals. This study reported common variant associations at 287 different genomic loci, and identified biological processes associated with the pathophysiology of SCZ. The findings also showed convergence in the association of SCZ with common and rare variants in neurodevelopmental disorders, which further provided a resource of prioritized genes and variants to advance mechanistic studies.

#### 2.1.2 GWAS dataset for Hypothyroidism

The data for hypothyroidism were obtained from FinnGen consortium R9 GWAS (https://r9.finngen.fi/), which included 40,926 cases and 274,069 controls. The following covariates were included in the model: sex, age, ten genetic principal components, and genotyping batch. The FinnGen study collected and analyzed genomic and health data from 500,000 Finnish Biobank participants. It not only provided novel medical and treatment-related insights, but also built a world-class resource that can be used for future research. See **Supplementary Table S1** for data sources and basic information. Data on thyroid function levels within the normal range were derived from a large meta-analysis of GWAS for thyroid function and dysfunction. The study tested 8 million genetic variants in up to 72,167 individuals, with a total of 109 independent genetic variants associated with these traits. The data for total TSH levels were derived from a GWAS meta-analysis of 22.4 million genetic markers from up to 119,715 individuals. The study also identified 74 genome-wide significant TSH loci.

### 2.2 Statistical analyses

#### 2.2.1 Genetic correlation analysis

Linkage disequilibrium score regression (LDSC) (35) and high-definition likelihood (HDL) (36) methods were used to assess genetic correlation among traits. The linkage disequilibrium (LD) score in the LDSC can be computed from a sample of European ancestry in the 1,000 Genomes Project that serves as a reference panel (37). The reference dataset for HDL was 1,029,876 quality-controlled HapMap3 single-nucleotide polymorphism (SNPs). For SNPs, we implemented strict quality control according to the following criteria: (i) we excluded non-bipartite allele SNPs and those with strand-ambiguous alleles; (ii) excluded SNPs without rs tags; (iii) deleted duplicated SNPs or SNPs that were not included in the 1000 Genomes Project or whose alleles were mismatched; (iv) due to their complex LD structure, SNPs located within the major histocompatibility complex region (chr6: 28.5–33.5Mb) were excluded in the LDSC analysis; (v) SNPs with minor allele frequency (MAF) > 0.01 were retained.

#### 2.2.2 PLACO

SNP-Level PLACO is a novel approach to studying pleiotropic loci between complex traits using only summary-level genotype-phenotype association statistics (38). We calculated the square of the *Z*-score for each variant and removed SNPs with very high *Z* ^2^ (>80). In addition, considering the potential correlation between complex diseases, we estimated a correlation matrix of the *Z*-score. The hypothesis of no pleiotropy was then tested using the horizontal α intersection union test (IUT) method. And the final *P* value of the IUT test was the maximum of the *P* values of tests H0 and H1.

Based on the PLACO results, we further mapped the identified motifs to nearby genes for exploring the common biological mechanisms of these pleiotropic loci. We performed a generalized gene-set analysis of GWAS Data using Multi-marker Analysis of GenoMic Annotation (MAGMA) (39) for genes located at or overlapping with pleiotropic loci based on PLACO outputs to identify pleiotropic candidate pathways, as well as the tissue enrichment of pleiotropic genes. By using given genetic data (e.g., GWAS data), MAGMA calculates an association score between each gene and a trait (e.g., disease). This involves combining genetic variants with gene annotations and weights to estimate the association between each gene and the trait. Functional mapping and annotation of genome-wide association studies (FUMA) were used to determine the biological functions of pleiotropic loci (40). A series of pathway enrichment analyses based on the Molecular Signatures Database (MSigDB) were used to determine the functions of mapped genes (41).

#### 2.2.3 MR analysis

We used the clumping program (42) in PLINK software to screen all significant loci independently associated with disease as instrumental variables (IVs) (*P<* 5×10^-8^), with the r^2^ threshold for IVs set at 0.001 and the window set at 10,000 kb. To ensure the strength of the IVs, we calculated the *r* ^2^ and *F* statistics for each instrumental variable (43). The F statistic is calculated as follows: $F=(\frac{n-1-k}{k})(\frac{r^{2}}{1-r^{2}})$ , where *r* ^2^ denotes the proportion of variance explained by the instrumental variable (IV), *n* represents the sample size, and *k* represents the number of SNPs. The main method used for MR is the inverse variance weighted (IVW) method, which requires that the IV satisfy three assumptions: (i) the IV should be correlated with the exposure; (ii) the IV should not be associated with confounders of the exposure and outcome associations; and (iii) the effect of the IV on the outcome is exclusively mediated by the exposure. We performed several sensitivity analyses. First, the Q-test using IVW and MR-Egger can detect potential violations of the assumptions through the heterogeneity of the associations between individual IVs (44). Second, we applied MR-Egger to estimate horizontal pleiotropy based on its intercept, ensuring that genetic variants were independently associated with exposure and outcome (45). We increased the stability and robustness of the results by using additional analyses [weighted median, weighted mode, debiased IVW (DIVW), MR-Robust Adjusted Profile Score (RAPS)] with different modeling assumptions and strengths of the MR method. Statistical analyses were performed in R3.5.3 software, and MR analyses were conducted using the MendelianRandomization package (46).

## 3 Results

### 3.1 Genetic correlation

Genetic correlation analysis revealed significant genetic correlations between hypothyroidism and ANX, MDD, and SCZ (**Table 1**), both in the LDSC and HDL approaches. The strongest correlations with MDD existed for hypothyroidism: LDSC (*r_g_* = 0.264, *P* = 7.35×10^-12^), HDL (*r_g_* = 0.304, *P* = 4.14×10^-17^). The intercept term of the LDSC regression excluded the possibility of sample overlap. In addition, HDL analysis of indicators related to thyroid function identified significant genetic correlations between MDD and FT4 levels with TSH levels.

**Table 1:** Genetic correlation between hypothyroidism and psychiatric disorders.

Trait pairs	LDSC			HDL	
	rg (SE)	*P*	Intercept (SE)	rg (SE)	*P*
Hypothyroidism & ANX	0.127 (0.056)	0.025	-0.006 (0.005)	0.125 (0.041)	0.003
Hypothyroidism & BIP	0.022 (0.032)	0.493	-0.002 (0.006)	-0.002 (0.022)	0.934
Hypothyroidism & MDD	0.264 (0.039)	7.35E-12	0.003 (0.005)	0.304 (0.036)	4.14E-17
Hypothyroidism & SCZ	0.057 (0.023)	0.016	0.002 (0.007)	0.08 (0.017)	3.89E-06
FT4 normal & ANX	0.126 (0.076)	0.097	-0.012 (0.005)	0.007 (0.049)	0.879
FT4 normal & BIP	-0.025 (0.041)	0.549	0.001 (0.005)	-0.025 (0.029)	0.399
FT4 normal & MDD	-0.063 (0.045)	0.161	0 (0.005)	-0.066 (0.032)	0.039
FT4 normal & SCZ	0.008 (0.028)	0.779	-0.002 (0.005)	0.008 (0.018)	0.675
TSH normal & ANX	-0.057 (0.078)	0.464	-0.001 (0.005)	-0.026 (0.044)	0.557
TSH normal & BIP	-0.048 (0.048)	0.322	0.003 (0.006)	-0.031 (0.023)	0.174
TSH normal & MDD	-0.052 (0.054)	0.338	-0.003 (0.005)	-0.059 (0.033)	0.074
TSH normal & SCZ	0.016 (0.036)	0.653	-0.008 (0.006)	-0.003 (0.017)	0.834
TSH total & ANX	-0.049 (0.059)	0.409	0.002 (0.005)	-0.02 (0.037)	0.591
TSH total & BIP	-0.055 (0.036)	0.128	0.008 (0.006)	-0.033 (0.023)	0.145
TSH total & MDD	-0.042 (0.042)	0.311	-0.003 (0.005)	-0.061 (0.027)	0.022
TSH total & SCZ	0.005 (0.028)	0.850	-0.003 (0.006)	-0.005 (0.018)	0.794

### 3.2 Identification and enrichment analysis of pleiotropic loci

**Figure 1:**
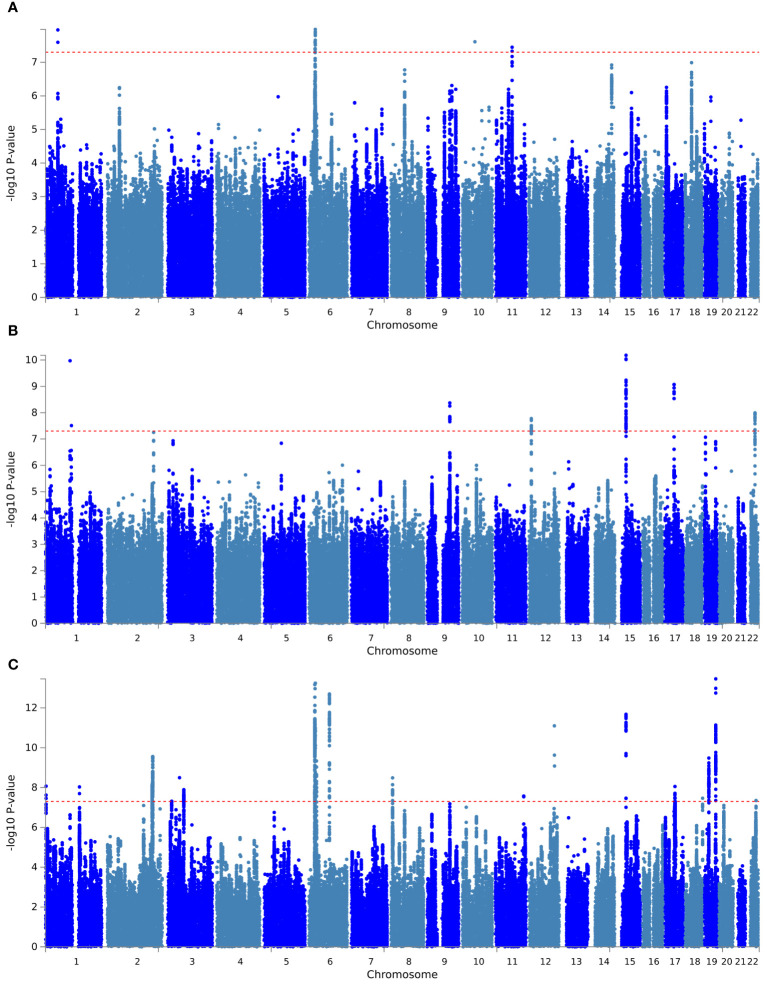
Manhattan plot of pleiotropic loci between hypothyroidism and psychiatric disorders. **(A)** Hypothyroidism versus MDD; **(B)** hypothyroidism versus ANX; **(C)** hypothyroidism versus SCZ. The red dashed line represents the significance level of 5×10^-8^.

First, PLACO pleiotropy analysis was performed between diseases, and the Manhattan plot was shown in **Figure 1**. A total of 30 pleiotropic risk loci were identified, which were shown in **Table 2**; **Supplementary Table S2**. Shared among different disease phenotypes at two loci. The 15q14 locus was identified simultaneously in ANX, SCZ diseases (*P* values were 6.59×10 ^-11^ and 2.10×10 ^-12^, respectively). The 6p22.1 locus was also identified in MDD, SCZ diseases (*P* values were 1.05E-08 and 5.75E-14, respectively). This analysis revealed a possible shared genetic basis between multiple diseases. The QQ plot did not identify genome inflation (**Supplementary Figure S1**), indicating that the results of our analysis have high statistical credibility. The basic information of each genomic risk locus was shown in **Supplementary Figure S2**, which detailed the size of each risk locus, the number of SNPs, the number of Map genes, and the number of genes located within the locus. The functional impact of pleiotropic SNPs on genes was shown in **Supplementary Figure S3**, and this section discusses in detail how specific SNP variants affect gene function and may consequently lead to alterations in disease risk. Among them, in hypothyroidism with MDD trait pair, the effect of pleiotropic SNPs on genes was mainly centered on intergenic. While, in hypothyroidism with ANX and SCZ trait pairs, the highest proportion was intronic. Further, PLACO pleiotropy analysis was performed between MDD and the two thyroid functions, and the Manhattan plot was shown in **Supplementary Figure S4**. A total of 16 pleiotropic risk loci were identified, which were shown in **Tables 2**; **Supplementary Figure S2**. Among them, four loci (1p32.3, 6p22.1, 10q21.1, 11q13.4) associated with MDD were identified in both FT4 normal and Hypothyroidism. This also revealed that MDD not only shares genes with hypothyroidism, but also has a genetic correlation with thyroid function. The QQ plot did not reveal genome inflation (**Supplementary Figure S5**), indicating that the results of our analysis have high statistical credibility. The basic information of each genomic risk loci was shown in **Supplementary Figure S6**, and the functional effects of pleiotropic SNPs on genes were shown in **Supplementary Figure S7**. As shown in the figure, in the MDD with FT4 normal trait pair, the effect of pleiotropic SNPs on genes was mainly focused on intergenic. While in the MDD with TSH total trait pair, the highest proportion was intronic.

**Table 2:** Information on the identified pleiotropic loci.

Trait pairs	Genomic Locus	Locus region	Lead SNP	P	Gene symbols
FT4 normal & MDD	1p32.3	1:54018849–54700034	rs72664111	1.09E-08	HNRNPA3P12, LDLRAD1
FT4 normal & MDD	6p22.1	6:27300310–29291002	rs853679	1.05E-08	ZSCAN31
FT4 normal & MDD	10q21.1	10:56829042–57303139	rs1857442	2.45E-08	PCDH15
FT4 normal & MDD	11q13.4	11:72946140–73731422	rs10751226	3.57E-08	FAM168A, AP000860.2
Hypothyroidism & ANX	1p13.3	1:108187943–108524977	rs7537605	1.07E-10	VAV3
Hypothyroidism & ANX	1p13.2	1:113871830–114651006	rs4839348	3.08E-08	RP4–590F24.1, SYT6
Hypothyroidism & ANX	9q22.33	9:100314613–100561486	rs10983513	4.25E-09	KRT18P13, RP11–546O6.4
Hypothyroidism & ANX	12p13.31	12:9443966–10046305	rs2268146	1.67E-08	CLECL1
Hypothyroidism & ANX	15q14	15:38817150–39009282	rs56059718	6.59E-11	RASGRP1
Hypothyroidism & ANX	17q21.2	17:40209533–40810228	rs13380830	8.49E-10	RAB5C, CTD-2132N18.3
Hypothyroidism & ANX	22q12.3	22:37571178–37675218	rs5845323	1.01E-08	C1QTNF6
Hypothyroidism & MDD	1p32.3	1:54018849–54700034	rs72664111	1.09E-08	HNRNPA3P12, LDLRAD1
Hypothyroidism & MDD	6p22.1	6:27300310–29291002	rs853679	1.05E-08	ZSCAN31
Hypothyroidism & MDD	10q21.1	10:56829042–57303139	rs1857442	2.45E-08	PCDH15
Hypothyroidism & MDD	11q13.4	11:72946140–73731422	rs10751226	3.57E-08	FAM168A, AP000860.2
Hypothyroidism & SCZ	1p36.32	1:2347837–2404401	rs4592207	8.52E-09	PLCH2
Hypothyroidism & SCZ	1q21.2	1:149237848–151021509	rs72692873	9.33E-09	OTUD7B, RP11–458I7.1
Hypothyroidism & SCZ	2q33.1	2:198144002–199166255	rs1595824	6.66E-09	PLCL1
Hypothyroidism & SCZ	2q33.1	2:200597298–201476430	rs143911669	2.77E-10	C2orf47, SPATS2L
Hypothyroidism & SCZ	3p24.3	3:17182233–17902583	rs62238360	4.86E-08	TBC1D5
Hypothyroidism & SCZ	3p21.1	3:52208046–53486737	rs731831	3.21E-09	STAB1
Hypothyroidism & SCZ	3p13	3:71329472–71688816	rs7624274	1.28E-08	FOXP1
Hypothyroidism & SCZ	6p22.1	6:25070838–29240378	rs1778482	5.75E-14	RP5–874C20.3
Hypothyroidism & SCZ	6p21.32	6:33179689–33780292	rs78306789	4.63E-10	ZBTB9, RN7SL26P
Hypothyroidism & SCZ	6q15	6:90806990–91089224	rs1010473	2.01E-13	BACH2
Hypothyroidism & SCZ	8p23.1	8:7129082–9091739	rs1878561	3.26E-09	FAM86B3P
Hypothyroidism & SCZ	11q24.2	11:124497410–124681679	rs55661361	2.67E-08	NRGN
Hypothyroidism & SCZ	12q24.12	12:111302295–113200293	rs35450384	7.99E-12	RP3–462E2.3, AC003029.1
Hypothyroidism & SCZ	15q14	15:38692498–38999345	rs12593201	2.10E-12	RASGRP1
Hypothyroidism & SCZ	17q21.31	17:43273992–44904147	rs62062288	8.87E-09	MAPT
Hypothyroidism & SCZ	18q23	18:77377921–77725683	rs71367544	3.45E-08	RP11–154H12.3
Hypothyroidism & SCZ	19p13.11	19:19260892–19961227	rs2905426	3.31E-10	MAU2, GATAD2A
Hypothyroidism & SCZ	19q13.33	19:49947383–50207165	rs7251	3.48E-14	IRF3
Hypothyroidism & SCZ	22q13.2	22:41397999–42741887	rs7290134	4.48E-08	TNFRSF13C
TSH total & MDD	1p36.13	1:19376373–19815320	rs12755497	4.55E-11	CAPZB
TSH total & MDD	1p31.3	1:61471237–61664841	rs384893	1.59E-09	NFIA
TSH total & MDD	2p21	2:43453086–43990725	rs6544658	3.72E-08	THADA
TSH total & MDD	3q29	3:193912078–193957929	rs74322585	5.21E-09	RP11–513G11.4
TSH total & MDD	5q13.3	5:76482340–76890387	rs7733908	1.33E-08	PDE8B
TSH total & MDD	6q27	6:165938582–166038611	rs3008011	2.98E-10	PDE10A
TSH total & MDD	9p24.2	9:4241674–4319392	rs6476842	4.23E-09	GLIS3
TSH total & MDD	9q22.33	9:100314613–100805464	rs1512261	3.13E-08	RP11–546O6.4, RP11–23B15.1
TSH total & MDD	12q23.1	12:96549543–96799409	rs78405390	3.01E-09	ELK3
TSH total & MDD	14q32.12	14:93432524–94032985	rs12879718	3.17E-14	ITPK1, RP11–371E8.2
TSH total & MDD	14q32.33	14:105151205–105277144	rs1132975	1.26E-08	SIVA1
TSH total & MDD	17q24.3	17:69739369–70173769	rs1966432	2.71E-09	SOX9-AS1

**Figure 2:**
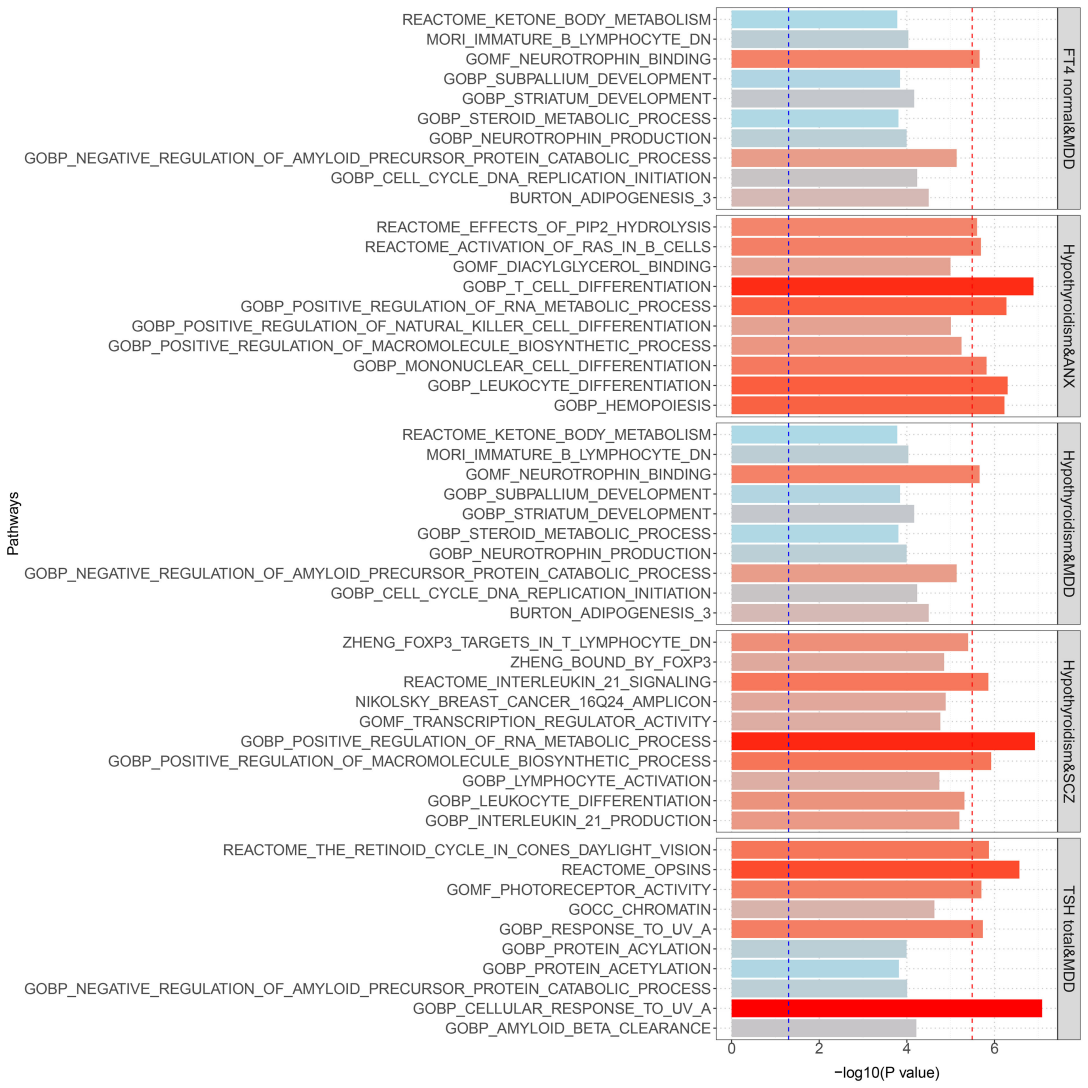
Results of genome-wide MAGMA gene set analysis (significant after multiple correction). The Blue dashed line is the 0.05 significance level; the red dashed line is the multiple-corrected significance level.

**Figure 3:**
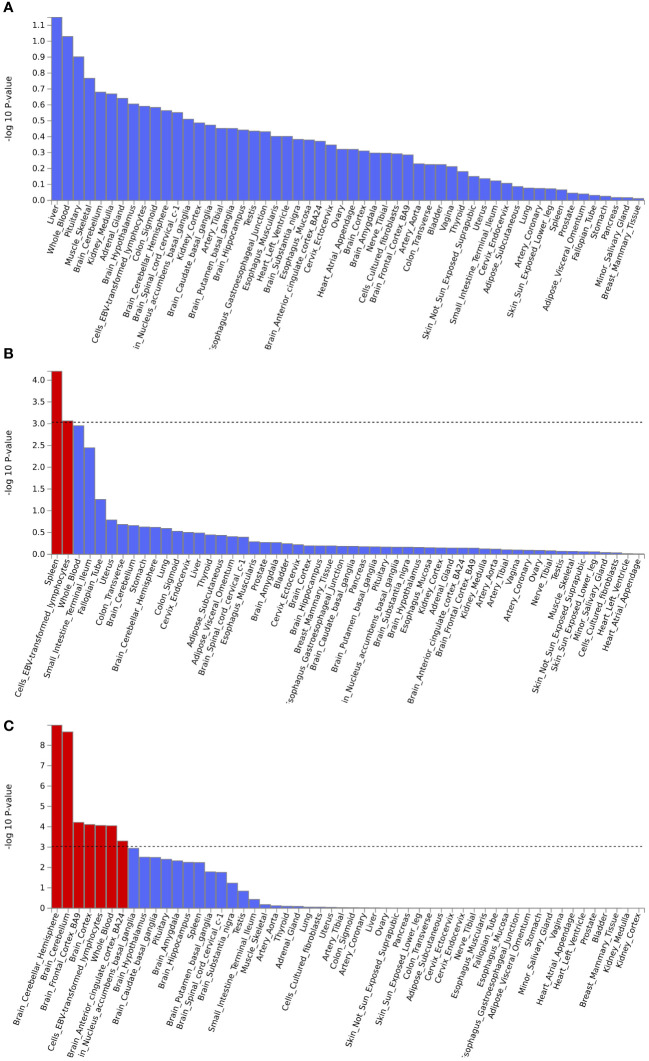
Enrichment analysis of pleiotropic effects in different tissues. **(A)** Hypothyroidism with MDD; **(B)** Hypothyroidism with ANX; **(C)** Hypothyroidism with SCZ. The red color represents significant results after multiple corrections.

Gene set enrichment analysis by MAGMA was performed on the pleiotropic results, which showed eleven significant gene sets that were enriched (**Figure 2**; **Supplementary Table S3**) involved multiple pathways, such as Cell response to UV-A, positive regulation of RNA metabolic process, T cell differentiation, reactome opsins, and so on. Tissue-specific MAGMA analysis showed that both diseases were significantly enriched in tissues such as brain and spleen (**Figure 3**; **Supplementary Figure S8**). Notably, this part of the MAGMA gene set and tissue-specific analyses were analyzed using the complete distribution of SNP *P* values.

Using the positional information of lead SNPs, we mapped nearby genes associated with these pleiotropic risk loci (**Supplementary Table S4**). The expression of position-matched pleiotropic genes in different tissues was shown in **Supplementary Figure S9**. Multiple genes were significantly differentially expressed in tissues such as the brain, whole blood, and lymphocytes. Tissue enrichment analysis revealed that these pleiotropic genes were significantly enriched in the adrenal gland (**Supplementary Figure S10**).

Further, MAGMA gene analysis identified 79 pleiotropic genes (*P*< 0.05/18776 = 2.66×10^-6^) (**Supplementary Figures S11**, **S13**, and **Supplementary Table S5**) with no genome inflation, which suggested that the results were credible (**Supplementary Figures S12**, **S14**). Multiple genes were shared among different disease pairs, such as ZNF462, VAV3, HIST1H2BN, ANP32B, PRMT1, B4GALT6, ZKSCAN4, BTN2A1, and ZSCAN16. Specific information on pleiotropic genes was summarized in **Supplementary Table S4**, and the expression of these pleiotropic genes in different tissues was shown in **Supplementary Figure S15**. The results revealed that these genes showed differential expression in some tissues, such as the brain, whole blood, lymphocytes, and other tissues. Tissue-specific enrichment analysis found that these genes were highly enriched into six brain-related tissues (**Supplementary Figure S16**), such as the anterior cingulate cortex, frontal cortex, basal ganglia, amygdala, brain cortex, and brain hippocampus.

**Figure 4:**
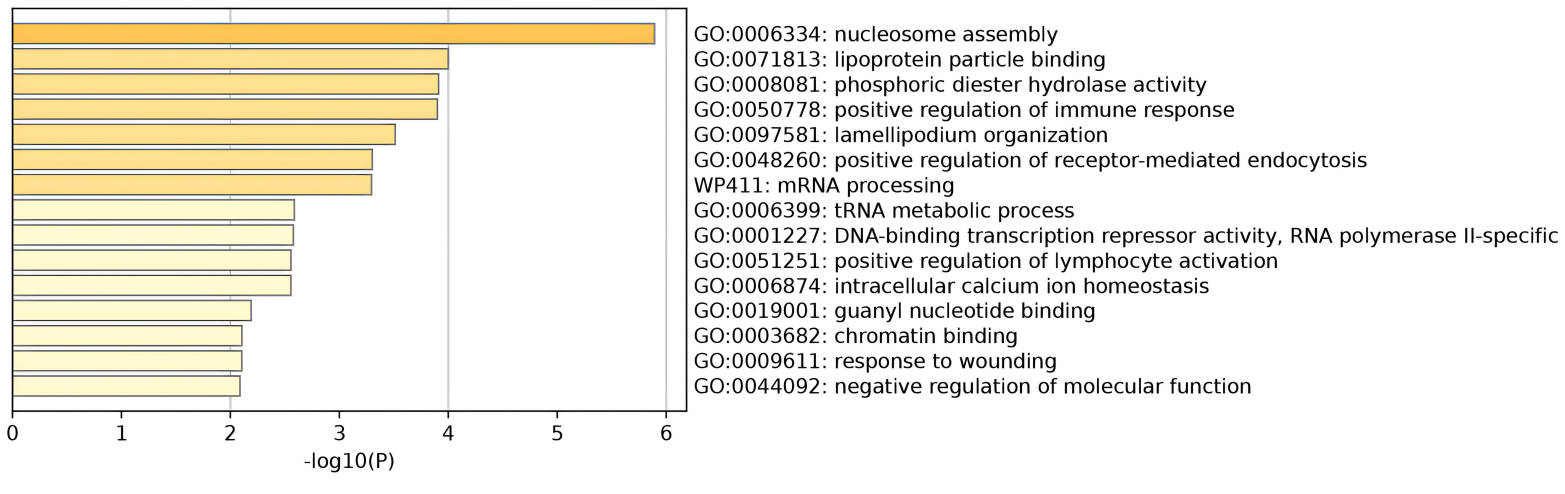
Pathway enrichment results for pleiotropic genes.

Pathway analyses were performed on nearby genes and MAGMA genes for pleiotropy. The results showed that nucleosome assembly and lipoprotein particle binding play important roles in thyroid function and psychiatric disorders. The main cell types enriched were adult renal tubular epithelial cells, skeletal muscle endothelial cells, midbrain neuronal cells, thymic vascular endothelial cells, and so on. Results were shown in **Figure 4**; **Supplementary Figure S17**. In addition, the protein-protein interaction network was constructed for the pleiotropic genes, and the key genes were screened. Eight key genes were screened, including MAPT, RAB2A, and others. As shown in **Supplementary Figure S18**. These proteins may play a role in different biological processes in cells, including gene expression regulation, cell signaling, cytoskeleton maintenance, etc.

### 3.3 Bidirectional two-sample MR analysis

Finally, we performed causal inference of the causal relationship between the two diseases using a bidirectional two-sample MR approach (**Table 3**). Instrumental variables were shown in **Supplementary Table S6**. The results supported the risk role of MDD for hypothyroidism. The analysis of the causal effect of MDD on Hypothyroidism showed that the three MR methods (IVW, DIVW, and MR-RAPS) presented consistent results, with MDD increasing the risk of hypothyroidism. The heterogeneity test ruled out the possibility of heterogeneity (*P*>0.05). The MR-Egger bias intercept term was *P*>0.05, so there was no effect of horizontal pleiotropy. Scatterplots and funnel plots ruled out the possibility of outlier interference.

**Table 3:** Results of significant causal association pairs.

Exposure	Outcome	Methods	Estimate (95%CI)	*P*	Heterogeneity test	
					Estimate	*P*
MDD	Hypothyroidism	IVW	1.43 (1.096, 1.866)	0.008	0.005	0.945
		DIVW	1.445 (1.083, 1.929)	0.012		
		MR-RAPS	1.43 (1.074, 1.905)	0.014		
BIP	TSH normal	IVW	-0.047 (-0.086, -0.007)	0.021	16.343	0.293
		DIVW	-0.048 (-0.091, -0.005)	0.027		
		MR-RAPS	-0.051 (-0.098, -0.004)	0.032		
		Weighted mode	-0.05 (-0.149, 0.05)	0.327		
		Weighted median	-0.048 (-0.105, 0.008)	0.094		
		MR-Egger (slope)	-0.166 (-0.444, 0.112)	0.262		
		MR-Egger (intercept)	0.011 (-0.014, 0.036)	0.409		

**Figure 5:**
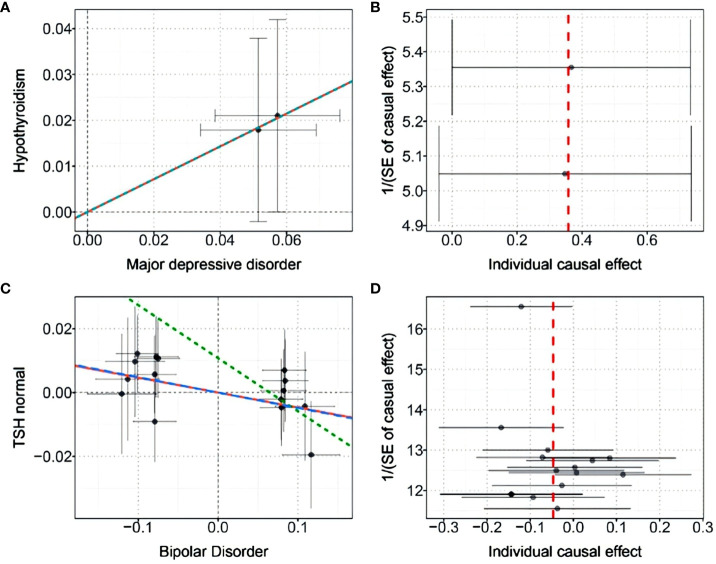
**(A)** Scatter plot of the causal effect of MDD on hypothyroidism; **(B)** Funnel plot of the causal effect of MDD on hypothyroidism; **(C)** Scatter plot of the causal effect of BIP on normal-range TSH levels; **(D)** Funnel plot of the causal effect of BIP on normal-range TSH levels.

The results also indicated that BIP could causally reduce TSH levels in the normal range. Analysis of the causal effect of BIP on TSH normal showed that the three MR methods (IVW, DIVW, and MR-RAPS) presented consistent results, with BIP reducing TSH normal levels. The heterogeneity test ruled out the possibility of heterogeneity (*P*>0.05). The MR-Egger bias intercept term was *P*>0.05, indicating that there was no effect of horizontal pleiotropy. Scatterplots and funnel plots ruled out the possibility of outlier interference. The scatter plots and funnel plots were shown in **Figure 5**.

GWAS data for hypothyroidism and thyroid function indicators were used as exposure factors, and GWAS data for psychiatric disorders were used as the outcomes for reverse MR analysis. After excluding SNPs with *P*>0.05 and removing chain imbalance, we failed to extract SNPs for the exposure factors in the outcome, i.e., there was no evidence of a causal effect between hypothyroidism or thyroid function indicators and psychiatric disorders. The results of all MR analyses and sensitivity analyses were shown in **Supplementary Table S7**.

## 4 Discussion

In this study, we found strong genetic correlations and genetic overlap between hypothyroidism and three psychiatric disorders (ANX, SCZ, and MDD) and identified common variants behind these associations. We also found strong genetic correlations between MDD and two thyroid functions (FT4 normal, TSH total). Further comprehensive analyses were performed to analyze the potential genetic basis of pleiotropic loci, pleiotropic genes, tissue enrichment, and biological pathways. Our two-sample MR analysis suggested that MDD has a significant causal effect on the increased risk of hypothyroidism and that BIP may reduce TSH normal level. These results advanced our understanding of the shared genetic architecture between hypothyroidism and psychiatric disorders and revealed a causal relationship between these traits, which suggested a common etiology and possible mechanisms for the coexistence of hypothyroidism and psychiatric disorders.

We first estimated the genetic correlation between hypothyroidism and psychiatric disorders, and identified a significant positive genetic correlation between hypothyroidism and ANX, MDD, and SCZ. This was consistent with previous observational studies, which found comorbidity between hypothyroidism and ANX, MDD, and SCZ (21, 26, 27). We found the strongest genetic correlation between hypothyroidism and MDD. TH may be the key to the link between the two. Previous studies have found that in the brain and nervous system, TH can regulate neurophysiological processes such as cell migration and differentiation, synaptogenesis, and myelination. These functions were strongly associated with emotional disorders such as depression and anxiety (47). Therefore, this was often used as an explanation for depression due to hypothyroidism. However, our two-sample MR results suggested that MDD may be responsible for the increased risk of hypothyroidism. It was found that antithyroid peroxidase (TPO) positivity was detected in the cerebrospinal fluid of patients with depression. This suggested that antithyroid antibodies were synthesized intrathecally in the central nervous system and may attack the thyroid gland either by direct action on nerve cells or by crossing the blood-brain barrier, leading to HT and subsequent hypothyroidism (48). Thus, depression may cause immune changes in the central nervous system thereby contributing to the progression of hypothyroidism. Based on the close correlation between the two, our findings supported the use of TH replacement therapy as an option for adjunctive treatment of drug-resistant depression (30). Another, our study did not find a genetic correlation between hypothyroidism and BIP, although previous studies suggested a comorbidity between the two (16). However, there may be a strong correlation between BIP and FT4 level. Previous studies have suggested that medications for BIP may lead to an increased risk of hypothyroidism. However, our MR results indicated a causal relationship between BIP and reduced FT4 level. These findings suggested that the monitoring of thyroid function should be emphasized in patients with BIP.

We identified multiple common risk loci for hypothyroidism and psychiatric disorders and identified 79 pleiotropic genes, several of which were shared among different disease pairs. We found that the 6p22.1 locus and genes, including ZKSCAN4 and MAPT, may play important roles in hypothyroidism and psychiatric disorders. Tissue enrichment revealed that these pleiotropic genes were highly enriched into six brain-related tissues. Chromosome 6p21-p22.1 spans the major histocompatibility complex (MHC) region and is a highly polymorphic, gene-dense region. Previous studies have identified it as a susceptibility locus for SCZ in Europeans, Japanese, and Chinese (49). Polymorphisms of zinc finger 4 (ZKSCAN4) with KRAB and SCAN structural domains located on chromosome 6p21-p22.1 were strongly associated with psychiatric disorders (49). MAPT has been found to be a causative protein in several psychiatric disorders and Parkinson’s disease. Mutations in the MAPT gene can lead to pathologic aggregation of tau proteins and death of glutamatergic cortex neurons (50, 51). Studies have also found significant genetic correlations between psychiatric disorders and structural brain phenotypes (cortical surface area, cortical thickness) (50). Brain function and structure have been found to be abnormal in patients with MDD. Studies have shown a major reduction in cerebral blood flow in the prefrontal cortex and anterior cingulate cortex in patients with MDD. Structural MRI studies have shown widespread reductions in brain volume of cortical and subcortical regions in patients with MDD, as well as reductions in the volume of the anterior cingulate cortex and hippocampus (52). On the other hand, TH plays a vital role in brain maturation and brain function throughout life (53). Meanwhile, it was found that TH receptors were widely distributed in several high-concentration areas of the brain, including the cerebral cortex, hippocampus, and amygdala, which were involved in the pathogenesis of psychiatric disorders (54).

We identified several enrichment pathways, all of which were involved to some extent in the pathogenesis of hypothyroidism and psychiatric disorders. Nucleosome assembly was a key epigenetic regulatory process involving the wrapping of DNA around histone octamers, which then affected gene expression (55). The location and assembly of nucleosomes have profound effects on gene expression. The tightness of nucleosome assembly can regulate the accessibility of specific gene regions, thereby affecting the binding of transcription factors and the activation or repression of genes (56). Our studies have revealed that pleiotropic loci may affect the expression or function of genes associated with nucleosome assembly, which collectively affect thyroid function and mental health. For example, a certain pleiotropic locus may lead to altered expression of histone variants, affecting the stability and dynamics of nucleosomes, which in turn affects the occurrence and development of related diseases. In thyroid disorders, such as hyper- or hypothyroidism, variations of nucleosome assembly may affect the expression of genes related to TH synthesis and metabolism. In psychiatric disorders, such as depression, changes in nucleosome assembly may affect the expression of genes related to neurotransmitter systems, including the serotonin delivery pathway (57). Lipoprotein particle binding involved the interaction of specific proteins with lipoproteins, such as low-density lipoprotein (LDL) or high-density lipoprotein (HDL) (58). These interactions were critical for lipid metabolism and hormone transport. The binding and transport functions of lipoprotein particles may play an important role in the distribution and regulation of TH (59, 60), influencing the cellular response to TH. There was an association between lipid metabolism and psychiatric disorders (e.g., depression, BID, etc.) (61). Variations of lipoprotein levels may be associated with neuroinflammation, neurotransmitter system function, and structural and functional changes in the brain (62). Lipoprotein particle binding may affect the transport and distribution of lipids in the brain, thereby affecting nervous system function and mental health (63).

Our study has several limitations. First, MR analysis was utilized to determine a causal relationship between hypothyroidism and the risk of psychiatric disorders. However, further biological studies and randomized controlled trials will be needed to validate the findings of this study. Second, our study mainly included participants of European ancestry and may not be generalizable to other ancestries. Therefore, further validation in other populations is necessary to demonstrate our results. However, there are few public GWAS summary data on thyroid function-related indices in other ethnicities.

## 5 Conclusions

This study provided an insight into the genetic and causal relationship between hypothyroidism and psychiatric disorders, which will help to understand the comorbidity of the disorders and their treatment. Our results showed significant genetic correlation between hypothyroidism and ANX, SCZ, and MDD. The shared loci, genes and pathways between hypothyroidism and psychiatric disorders provided insights into their comorbidity, genetic causes, and biological mechanisms. In addition, we observed a causal relationship between MDD and hypothyroidism and between BIP and FT4 levels. In this study, we obtained GWAS pooled data mainly from Europe. In the future, we aim to further analyze other populations.

## Data availability statement

The original contributions presented in the study are included in the article/**Supplementary Material**. Further inquiries can be directed to the corresponding author.

## Author contributions

JZ: Conceptualization, Data curation, Formal analysis, Investigation, Methodology, Project administration, Software, Visualization, Writing – original draft. LZ: Conceptualization, Data curation, Formal analysis, Investigation, Project administration, Software, Supervision, Writing – review & editing.

## References

[1] Zhao X Cao Y Jin H Wang X Zhang L Zhang Y. Hydrogen sulfide promotes thyroid hormone synthesis and secretion by upregulating sirtuin-1. Front Pharmacol. (2022) 13:838248. doi: 10.3389/fphar.2022.838248 35222046 PMC8866871

[2] Wang Y Li Q Yuan Z Ma S Shao S Wu Y. Statin use and benefits of thyroid function: A retrospective cohort study. Front Endocrinol (Lausanne). (2021) 12:578909. doi: 10.3389/fendo.2021.578909 33737906 PMC7962670

[3] Syed W Samarkandi OA Alsadoun A Harbi MKA Al-Rawi MBA. Evaluation of clinical knowledge and perceptions about the development of thyroid cancer-An observational study of healthcare undergraduates in Saudi Arabia. Front Public Health. (2022) 10:912424. doi: 10.3389/fpubh.2022.912424 36052013 PMC9426299

[4] Li Y Teng D Ba J Chen B Du J He L. Efficacy and safety of long-term universal salt iodization on thyroid disorders: epidemiological evidence from 31 provinces of mainland China. Thyroid. (2020) 30:568–79. doi: 10.1089/thy.2019.0067 32075540

[5] Garber JR Cobin RH Gharib H Hennessey JV Klein I Mechanick JI. Clinical practice guidelines for hypothyroidism in adults: cosponsored by the American Association of Clinical Endocrinologists and the American Thyroid Association. Endocr Pract. (2012) 18:988–1028. doi: 10.4158/EP12280.GL 23246686

[6] Teumer A Chaker L Groeneweg S Li Y Di Munno C Barbieri C. Genome-wide analyses identify a role for SLC17A4 and AADAT in thyroid hormone regulation. Nat Commun. (2018) 9:4455. doi: 10.1038/s41467-018-06356-1 30367059 PMC6203810

[7] Meling Stokland AE Ueland G Lima K Grønning K Finnes TE Svendsen M. Autoimmune thyroid disorders in autoimmune addison disease. J Clin Endocrinol Metab. (2022) 107:e2331–e8. doi: 10.1210/clinem/dgac089 PMC9113809 35226748

[8] Xiong S Peng H Ding X Wang X Wang L Wu C. Circular RNA Expression Profiling and the Potential Role of hsa_circ_0089172 in Hashimoto's Thyroiditis *via* Sponging miR125a-3p. Mol Ther Nucleic Acids. (2019) 17:38–48. doi: 10.1016/j.omtn.2019.05.004 31207490 PMC6579753

[9] Tabasi F Hasanpour V Sarhadi S Kaykhaei MA Pourzand P Heravi M. Association of miR-499 polymorphism and its regulatory networks with hashimoto thyroiditis susceptibility: a population-based case-control study. Int J Mol Sci. (2021) 22(18):10094. doi: 10.3390/ijms221810094 34576267 PMC8470033

[10] Li N Gao S Wang S He S Wang J He L. Attractin participates in schizophrenia by affecting testosterone levels. Front Cell Dev Biol. (2021) 9:755165. doi: 10.3389/fcell.2021.755165 34869343 PMC8636034

[11] Charlson FJ Baxter AJ Dua T Degenhardt L Whiteford HA Vos T. Excess mortality from mental, neurological, and substance use disorders in the global burden of disease study 2010. In: Patel V Chisholm D Dua T Laxminarayan R Medina-Mora ME, editors. Mental, neurological, and substance use disorders: disease control priorities, 3rd ed., vol. 4 . The International Bank for Reconstruction and Development / The World Bank © 2016 International Bank for Reconstruction and Development / The World Bank, Washington (DC (2016). 27227239

[12] Murray LK Haroz E Dorsey S Kane J Bolton PA Pullmann MD. Understanding mechanisms of change: An unpacking study of the evidence-based common-elements treatment approach (CETA) in low and middle income countries. Behav Res Ther. (2020) 130:103430. doi: 10.1016/j.brat.2019.103430 31780251 PMC8114793

[13] Walker ER McGee RE Druss BG. Mortality in mental disorders and global disease burden implications: a systematic review and meta-analysis. JAMA Psychiatry. (2015) 72:334–41. doi: 10.1001/jamapsychiatry.2014.2502 PMC4461039 25671328

[14] Zhang W Sweeney JA Yao L Li S Zeng J Xu M. Brain structural correlates of familial risk for mental illness: a meta-analysis of voxel-based morphometry studies in relatives of patients with psychotic or mood disorders. Neuropsychopharmacology. (2020) 45:1369–79. doi: 10.1038/s41386-020-0687-y PMC7297956 32353861

[15] Koskinen MK Hovatta I. Genetic insights into the neurobiology of anxiety. Trends Neurosci. (2023) 46:318–31. doi: 10.1016/j.tins.2023.01.007 36828693

[16] Lekurwale V Acharya S Shukla S Kumar S. Neuropsychiatric manifestations of thyroid diseases. Cureus. (2023) 15:e33987. doi: 10.7759/cureus.33987 36811059 PMC9938951

[17] Noda M. Possible role of glial cells in the relationship between thyroid dysfunction and mental disorders. Front Cell Neurosci. (2015) 9:194. doi: 10.3389/fncel.2015.00194 26089777 PMC4452882

[18] Morley JE Shafer RB. Thyroid function screening in new psychiatric admissions. Arch Intern Med. (1982) 142:591–3. doi: 10.1001/archinte.142.3.591 7065794

[19] Wu SQ Feng F Zou RJ Fu HL Sun JW Jia XZ. Abnormal brain glucose metabolism in papillary thyroid cancer patients 4 weeks after withdrawal of levothyroxine: A cross-sectional study using (18)F-FDG PET/CT. Front Endocrinol (Lausanne). (2021) 12:595933. doi: 10.3389/fendo.2021.595933 33776909 PMC7992039

[20] Wu J Wang Z Xu H Yang L Liu J Zheng Y. Thyroid dysfunction in young, first-episode and drug-naïve patients with major depressive disorder: prevalence and associated clinical factors. Front Psychiatry. (2023) 14:1156481. doi: 10.3389/fpsyt.2023.1156481 37457778 PMC10348838

[21] Sharif K Tiosano S Watad A Comaneshter D Cohen AD Shoenfeld Y. The link between schizophrenia and hypothyroidism: a population-based study. Immunol Res. (2018) 66:663–7. doi: 10.1007/s12026-018-9030-7 30350120

[22] Gyllenberg D Sourander A Surcel HM Hinkka-Yli-Salomäki S McKeague IW Brown AS. Hypothyroxinemia during gestation and offspring schizophrenia in a national birth cohort. Biol Psychiatry. (2016) 79:962–70. doi: 10.1016/j.biopsych.2015.06.014 PMC4684794 26194598

[23] Caturegli P De Remigis A Chuang K Dembele M Iwama A Iwama S. Hashimoto's thyroiditis: celebrating the centennial through the lens of the Johns Hopkins hospital surgical pathology records. Thyroid. (2013) 23:142–50. doi: 10.1089/thy.2012.0554 PMC3569966 23151083

[24] Spann MN Cheslack-Postava K Brown AS. The association of serologically documented maternal thyroid conditions during pregnancy with bipolar disorder in offspring. Bipolar Disord. (2020) 22:621–8. doi: 10.1111/bdi.12879 31758834

[25] Hirtz R Föcker M Libuda L Antel J Öztürk D Kiewert C. Increased prevalence of subclinical hypothyroidism and thyroid autoimmunity in depressed adolescents: results from a clinical cross-sectional study in comparison to the general pediatric population. J Clin Psychiatry. (2021) 82(2):20m13511. doi: 10.4088/JCP.20m13511 33989468

[26] Siegmann EM Müller HHO Luecke C Philipsen A Kornhuber J Grömer TW. Association of depression and anxiety disorders with autoimmune thyroiditis: A systematic review and meta-analysis. JAMA Psychiatry. (2018) 75:577–84. doi: 10.1001/jamapsychiatry.2018.0190 PMC6137529 29800939

[27] Ittermann T Völzke H Baumeister SE Appel K Grabe HJ. Diagnosed thyroid disorders are associated with depression and anxiety. Soc Psychiatry Psychiatr Epidemiol. (2015) 50:1417–25. doi: 10.1007/s00127-015-1043-0 25777685

[28] Engum A Bjøro T Mykletun A Dahl AA. An association between depression, anxiety and thyroid function–a clinical fact or an artefact? Acta Psychiatr Scand. (2002) 106:27–34. doi: 10.1034/j.1600-0447.2002.01250.x 12100345

[29] Klubo-Gwiezdzinska J Wartofsky L. Hashimoto thyroiditis: an evidence-based guide to etiology, diagnosis and treatment. Pol Arch Intern Med. (2022) 132(3):16222. doi: 10.20452/pamw.16222 35243857 PMC9478900

[30] Głombik K Detka J Budziszewska B. Venlafaxine and L-thyroxine treatment combination: impact on metabolic and synaptic plasticity changes in an animal model of coexisting depression and hypothyroidism. Cells.. (2021) 10(6):1394. doi: 10.3390/cells10061394 34198731 PMC8227539

[31] Zhao Y Wen SW Li M Sun Z Yuan X Retnakaran R. Dose-response association of acute-phase quetiapine treatment with risk of new-onset hypothyroidism in schizophrenia patients. Br J Clin Pharmacol. (2021) 87:4823–30. doi: 10.1111/bcp.14928 34046922

[32] Bou Khalil R Richa S. Thyroid adverse effects of psychotropic drugs: a review. Clin Neuropharmacol. (2011) 34:248–55. doi: 10.1097/WNF.0b013e31823429a7 21996646

[33] Paunović VR Timotijević I Marinković D. Neuroleptic actions on the thyroid axis: different effects of clozapine and haloperidol. Int Clin Psychopharmacol. (1991) 6:133–9. doi: 10.1097/00004850-199100630-00001 1806619

[34] Nederlof M Kupka RW Braam AM Egberts A Heerdink ER. Evaluation of clarity of presentation and applicability of monitoring instructions for patients using lithium in clinical practice guidelines for treatment of bipolar disorder. Bipolar Disord. (2018) 20:708–20. doi: 10.1111/bdi.12681 PMC6585994 30105767

[35] Bulik-Sullivan B Finucane HK Anttila V Gusev A Day FR Loh PR. An atlas of genetic correlations across human diseases and traits. Nat Genet. (2015) 47:1236–41. doi: 10.1038/ng.3406 PMC4797329 26414676

[36] Ning Z Pawitan Y Shen X. High-definition likelihood inference of genetic correlations across human complex traits. Nat Genet. (2020) 52:859–64. doi: 10.1038/s41588-020-0653-y 32601477

[37] Auton A Brooks LD Durbin RM Garrison EP Kang HM Korbel JO. A global reference for human genetic variation. Nature. (2015) 526:68–74. doi: 10.1038/nature15393 26432245 PMC4750478

[38] Ray D Chatterjee N. A powerful method for pleiotropic analysis under composite null hypothesis identifies novel shared loci between Type 2 Diabetes and Prostate Cancer. PloS Genet. (2020) 16:e1009218. doi: 10.1371/journal.pgen.1009218 33290408 PMC7748289

[39] de Leeuw CA Mooij JM Heskes T Posthuma D. MAGMA: generalized gene-set analysis of GWAS data. PloS Comput Biol. (2015) 11:e1004219. doi: 10.1371/journal.pcbi.1004219 25885710 PMC4401657

[40] Watanabe K Taskesen E van Bochoven A Posthuma D. Functional mapping and annotation of genetic associations with FUMA. Nat Commun. (2017) 8:1826. doi: 10.1038/s41467-017-01261-5 29184056 PMC5705698

[41] Subramanian A Tamayo P Mootha VK Mukherjee S Ebert BL Gillette MA. Gene set enrichment analysis: A knowledge-based approach for interpreting genome-wide expression profiles. Proc Natl Acad Sci. (2005) 102:15545–50. doi: 10.1073/pnas.0506580102 PMC1239896 16199517

[42] Purcell S Neale B Todd-Brown K Thomas L Ferreira MA Bender D. PLINK: a tool set for whole-genome association and population-based linkage analyses. Am J Hum Genet. (2007) 81:559–75. doi: 10.1086/519795 PMC1950838 17701901

[43] Burgess S Thompson SG. Avoiding bias from weak instruments in Mendelian randomization studies. Int J Epidemiol. (2011) 40:755–64. doi: 10.1093/ije/dyr036 21414999

[44] Thompson SG Sharp SJ. Explaining heterogeneity in meta-analysis: A comparison of methods. Stat Med. (1999) 18:2693–708. doi: 10.1002/(SICI)1097-0258(19991030)18:20<2693::AID-SIM235>3.3.CO;2-M 10521860

[45] Burgess S Thompson SG. Interpreting findings from Mendelian randomization using the MR-Egger method. Eur J Epidemiol. (2017) 32:377–89. doi: 10.1007/s10654-017-0255-x PMC5506233 28527048

[46] Yavorska OO Burgess S. MendelianRandomization: an R package for performing Mendelian randomization analyses using summarized data. Int J Epidemiol. (2017) 46:1734–9. doi: 10.1093/ije/dyx034 PMC5510723 28398548

[47] Baksi S Pradhan A. Thyroid hormone: sex-dependent role in nervous system regulation and disease. Biol Sex Differ. (2021) 12:25. doi: 10.1186/s13293-021-00367-2 33685490 PMC7971120

[48] Dersch R Tebartz van Elst L Hochstuhl B Fiebich BL Stich O Robinson T. Anti-thyroid peroxidase and anti-thyroglobulin autoantibodies in the cerebrospinal fluid of patients with unipolar depression. J Clin Med. (2020) 9(8):2391. doi: 10.3390/jcm9082391 32726952 PMC7465032

[49] Zhang Y Lu T Yan H Ruan Y Wang L Zhang D. Replication of association between schizophrenia and chromosome 6p21-6p22.1 polymorphisms in Chinese Han population. PloS One (2013) 8:e56732. doi: 10.1371/journal.pone.0056732 23437227 PMC3578928

[50] Wingo TS Liu Y Gerasimov ES Vattathil SM Wynne ME Liu J. Shared mechanisms across the major psychiatric and neurodegenerative diseases. Nat Commun. (2022) 13:4314. doi: 10.1038/s41467-022-31873-5 35882878 PMC9325708

[51] Ruiz-Gabarre D Vallés-Saiz L Carnero-Espejo A Ferrer I Hernández F Garcia-Escudero R. Intron retention as a productive mechanism in human MAPT: RNA species generated by retention of intron 3. EBioMedicine. (2024) 100:104953. doi: 10.1016/j.ebiom.2023.104953 38181704 PMC10789595

[52] Vasic N Wolf ND Grön G Sosic-Vasic Z Connemann BJ Sambataro F. Baseline brain perfusion and brain structure in patients with major depression: a multimodal magnetic resonance imaging study. J Psychiatry Neurosci. (2015) 40:412–21. doi: 10.1503/jpn PMC4622640 26125119

[53] Singh S Yazdani U Gadad B Zaman S Hynan LS Roatch N. Serum thyroid-stimulating hormone and interleukin-8 levels in boys with autism spectrum disorder. J Neuroinflammation. (2017) 14:113. doi: 10.1186/s12974-017-0888-4 28577577 PMC5457729

[54] Williams GR. Neurodevelopmental and neurophysiological actions of thyroid hormone. J Neuroendocrinol. (2008) 20:784–94. doi: 10.1111/j.1365-2826.2008.01733.x 18601701

[55] North JA Šimon M Ferdinand MB Shoffner MA Picking JW Howard CJ. Histone H3 phosphorylation near the nucleosome dyad alters chromatin structure. Nucleic Acids Res. (2014) 42:4922–33. doi: 10.1093/nar/gku150 PMC4005658 24561803

[56] Zhao Y Wang J Liang F Liu Y Wang Q Zhang H. NucMap: a database of genome-wide nucleosome positioning map across species. Nucleic Acids Res. (2019) 47:D163–d9. doi: 10.1093/nar/gky980 PMC6323900 30335176

[57] Wu X Ding M Liu Y Xia X Xu FL Yao J. hsa-miR-3177-5p and hsa-miR-3178 Inhibit 5-HT1A Expression by Binding the 3'-UTR Region *in vitro* . Front Mol Neurosci. (2019) 12:13. doi: 10.3389/fnmol.2019.00013 30766477 PMC6365703

[58] Egom EE Fitzgerald R Canning R Pharithi RB Murphy C Maher V. Determination of sphingosine-1-phosphate in human plasma using liquid chromatography coupled with Q-tof mass spectrometry. Int J Mol Sci. (2017) 18(8):1800. doi: 10.3390/ijms18081800 28820460 PMC5578187

[59] Omar AE Al-Khalaifah HS Osman A Gouda A Shalaby SI Roushdy EM. Modulating the growth, antioxidant activity, and immunoexpression of proinflammatory cytokines and apoptotic proteins in broiler chickens by adding dietary spirulina platensis phycocyanin. Antioxidants (Basel). (2022) 11(5):991. doi: 10.3390/antiox11050991 35624855 PMC9137683

[60] Somppi TL. Non-thyroidal illness syndrome in patients exposed to indoor air dampness microbiota treated successfully with triiodothyronine. Front Immunol. (2017) 8:919. doi: 10.3389/fimmu.2017.00919 28824644 PMC5545575

[61] Lesiewska N Borkowska A Junik R Kamińska A Pulkowska-Ulfig J Tretyn A. The association between affective temperament traits and dopamine genes in obese population. Int J Mol Sci. (2019) 20(8):1847. doi: 10.3390/ijms20081847 30991630 PMC6515197

[62] Warstadt NM Dennis EL Jahanshad N Kohannim O Nir TM McMahon KL. Serum cholesterol and variant in cholesterol-related gene CETP predict white matter microstructure. Neurobiol Aging. (2014) 35:2504–13. doi: 10.1016/j.neurobiolaging.2014.05.024 PMC4198330 24997672

[63] Yang D Wang X Zhang L Fang Y Zheng Q Liu X. Lipid metabolism and storage in neuroglia: role in brain development and neurodegenerative diseases. Cell Biosci. (2022) 12:106. doi: 10.1186/s13578-022-00828-0 35831869 PMC9277953

