# Supplementary material for: Shared genetic links between hypothyroidism and psychiatric disorders: evidence from a comprehensive genetic analysis
Source: Front Endocrinol (Lausanne). 2024 Jun 6;15:1370019. doi: 10.3389/fendo.2024.1370019 (PMC11187243; doi:10.3389/fendo.2024.1370019)
Supplement: Supplementary file 2 [file DataSheet_2.docx]

**Supplementary Figures 1-18**

**
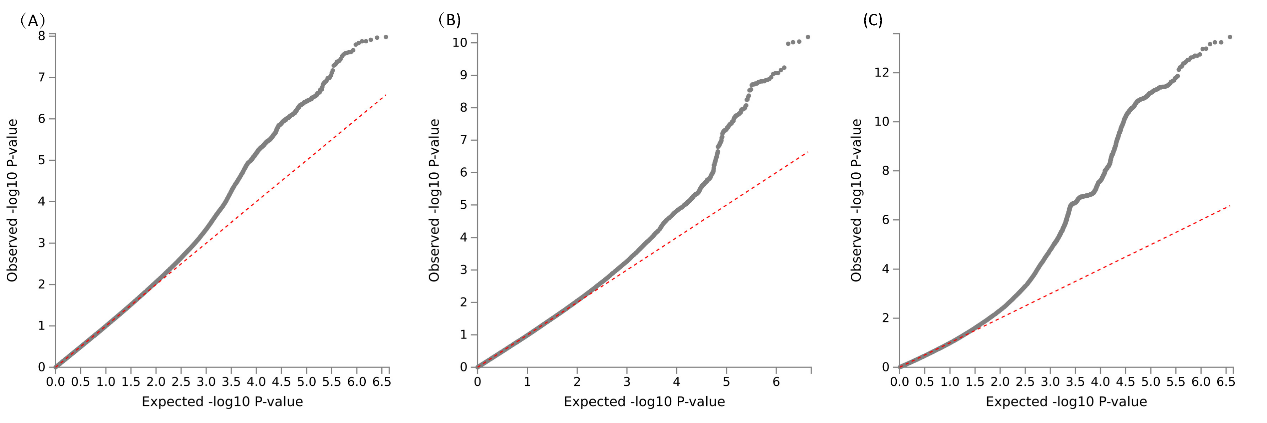
**

**Figure S1.** QQ plot of pleiotropic analysis between hypothyroidism and psychiatric disorders. (A) Hypothyroidism and MDD; (B) Hypothyroidism and ANX; (C) Hypothyroidism and SCZ.


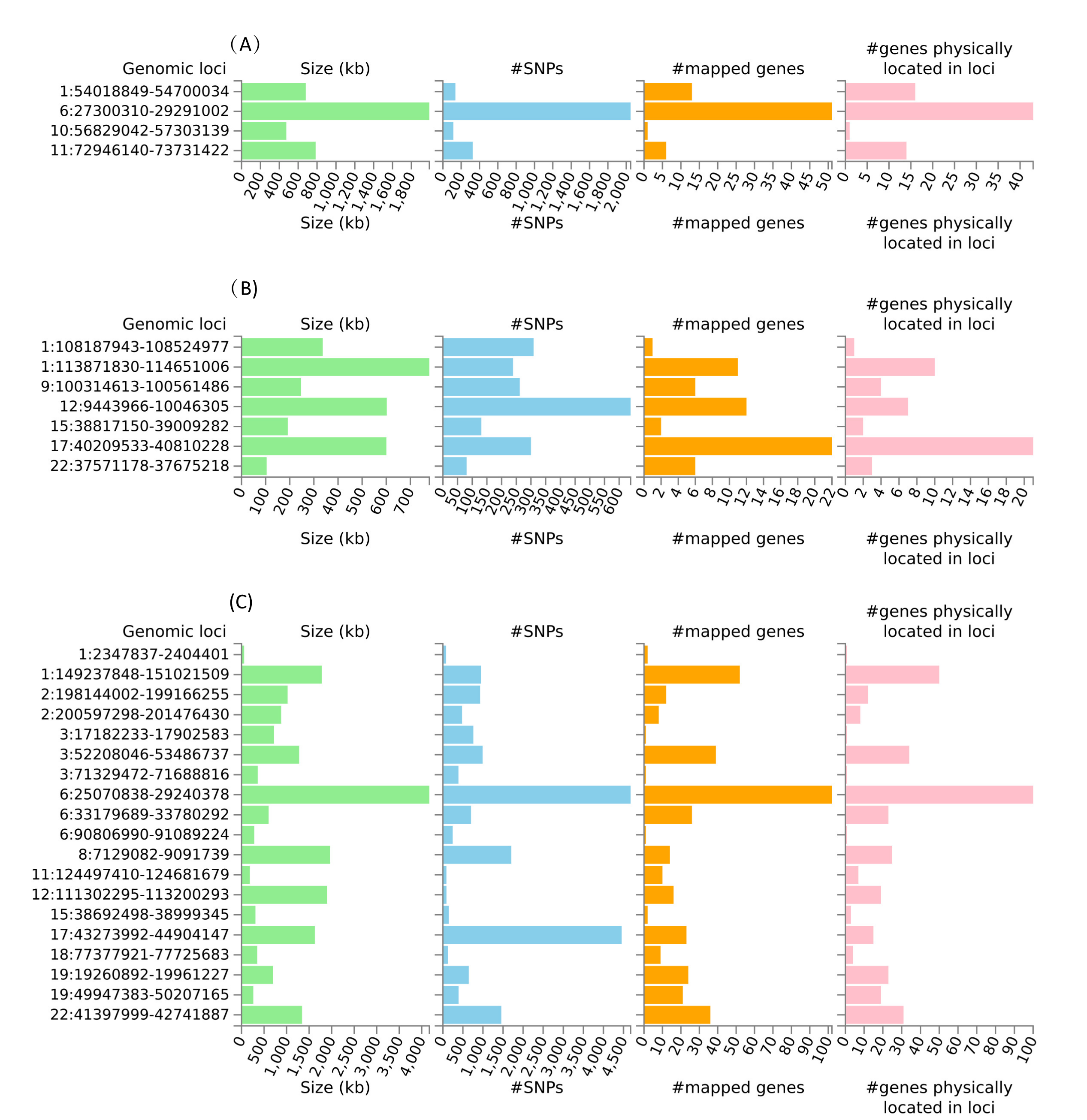


**Figure S2.** Summary of basic information for each genomic risk loci. (A) Hypothyroidism and MDD; (B) Hypothyroidism and ANX; (C) Hypothyroidism and SCZ. (From left to right, size of the risk loci, number of SNPs, number of Map genes, and number of genes located within the loci, respectively.)


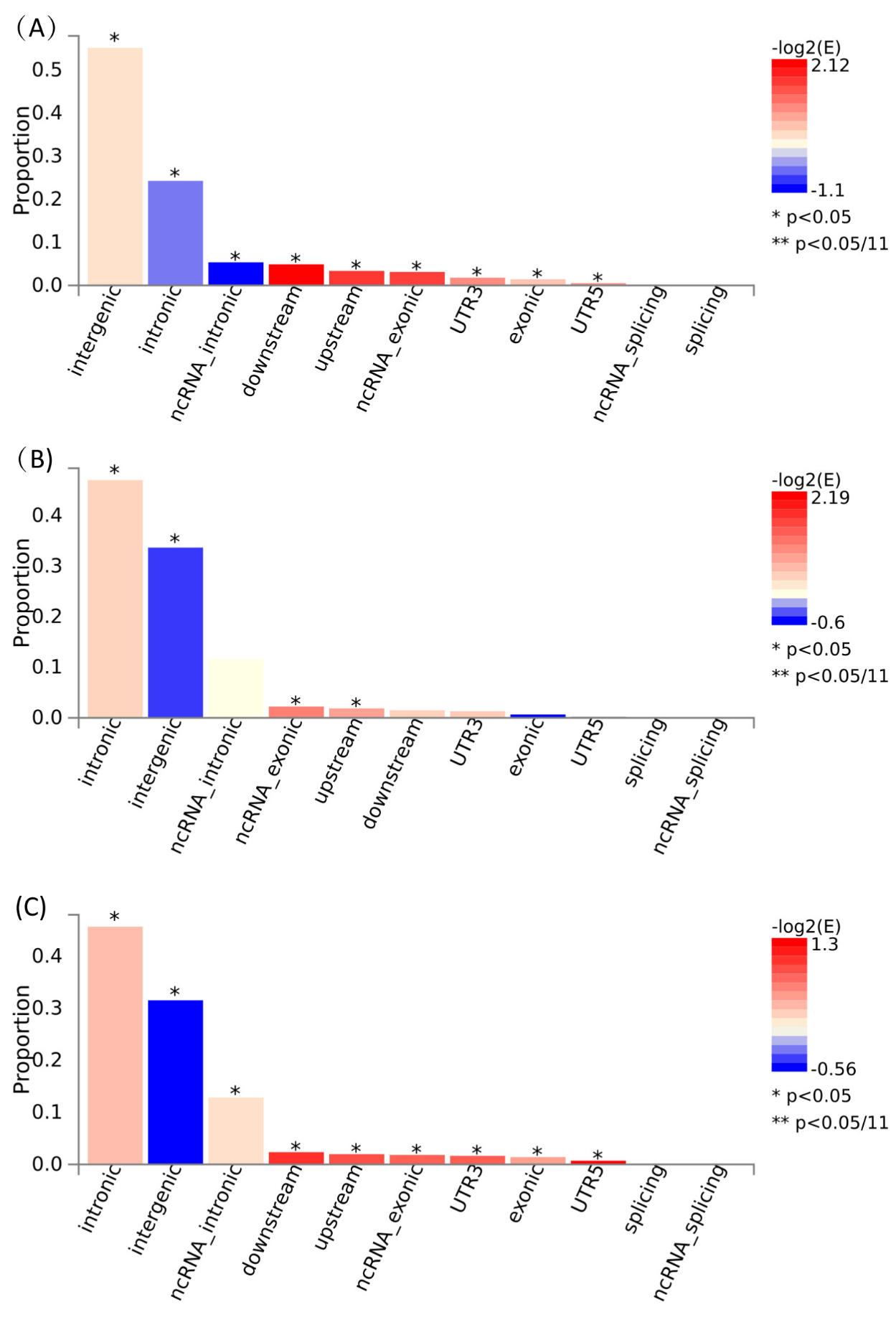


**Figure S3.** Functional effects of pleiotropic SNPs on genes. (A) Hypothyroidism and MDD; (B) Hypothyroidism and ANX; (C) Hypothyroidism and SCZ.


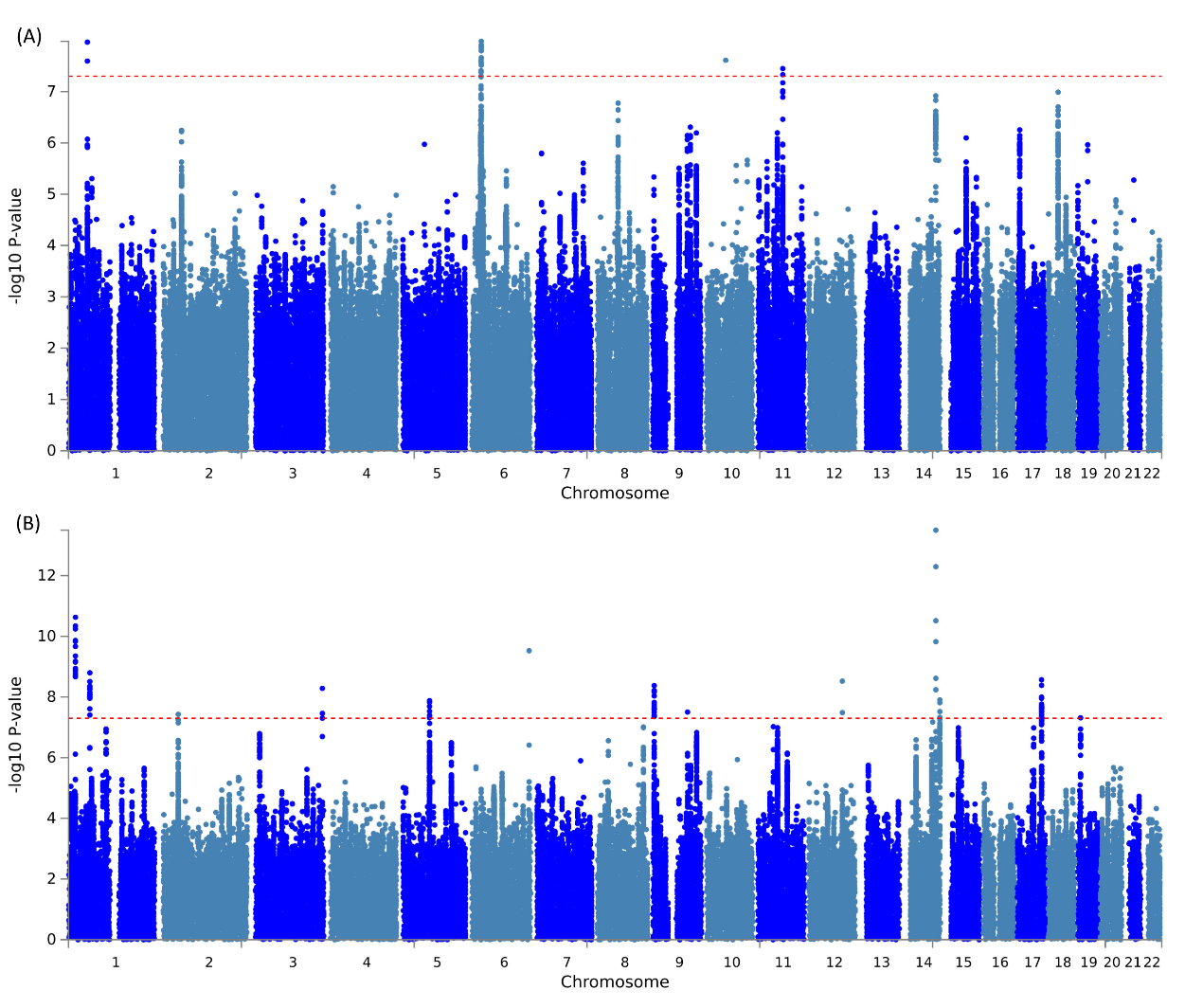


**Figure S4.** Manhattan plots of pleiotropic loci between thyroid function and MDD. (A) MDD and FT4 normal; (B) MDD and TSH total. The red dashed line represents the significance level of 5×10^-8^.


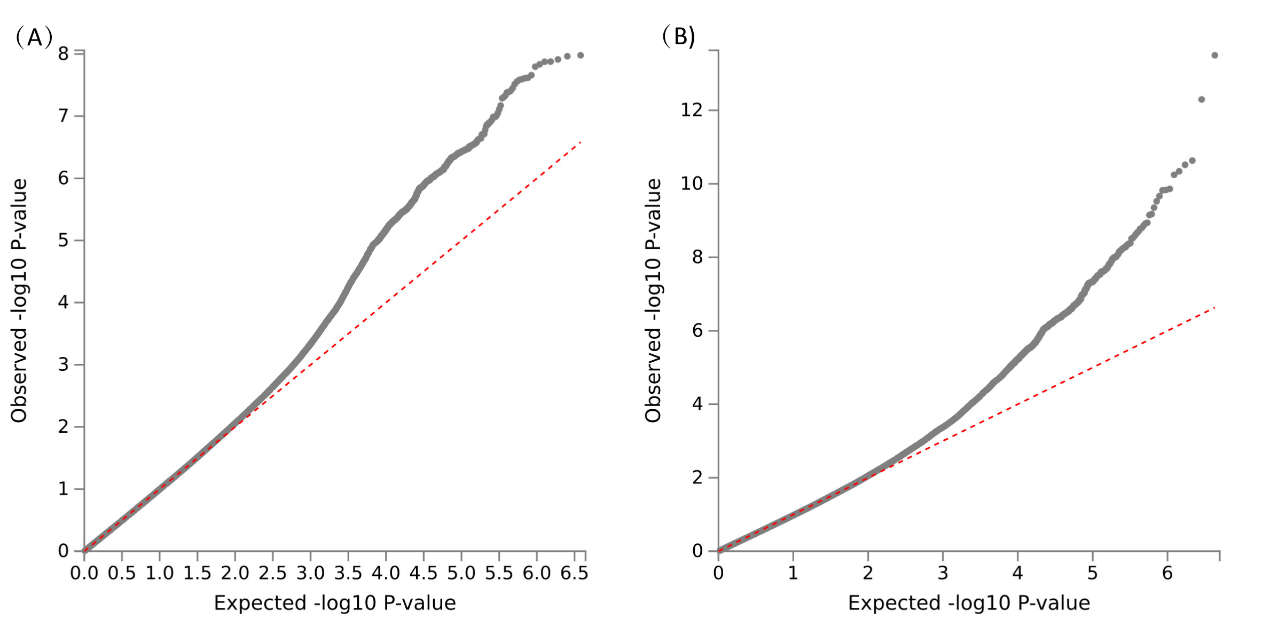


**Figure S5.** QQ plot on the pleiotropic analysis of thyroid function and MDD. (A) MDD and FT4 normal; (B) MDD and TSH total.


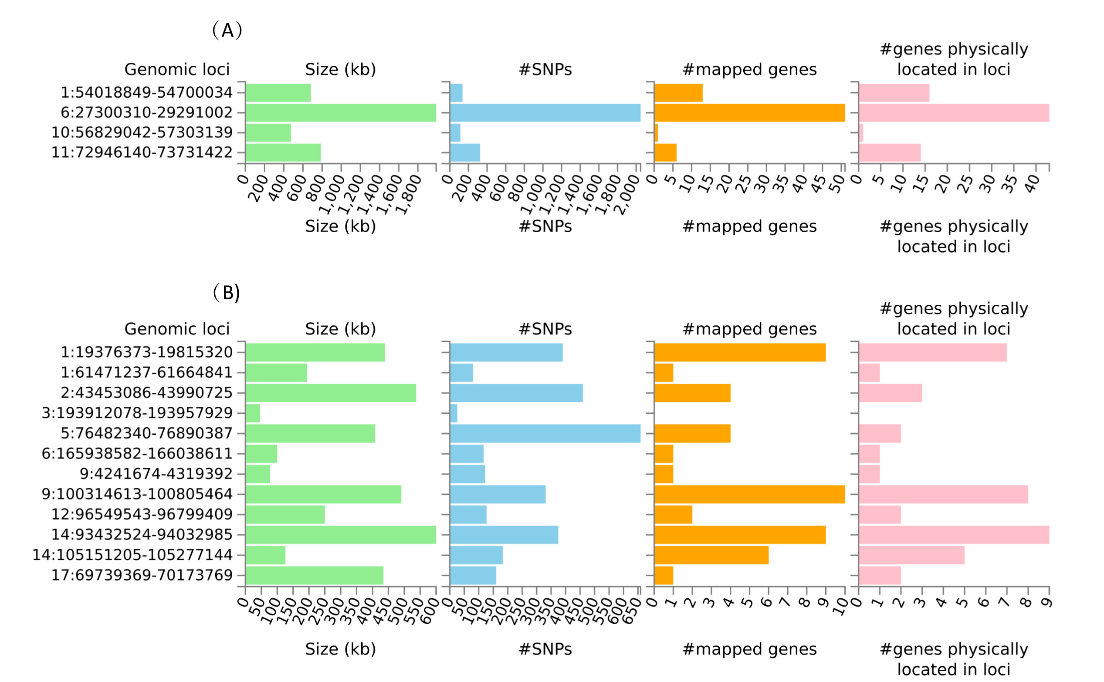


**Figure S6.** Summary of basic information for each genomic risk locus. (A) MDD and FT4 normal; (B) MDD and TSH total. (From left to right, the size of the risk loci, the number of SNPs, the number of Map genes, and the number of genes located within the loci, respectively.)


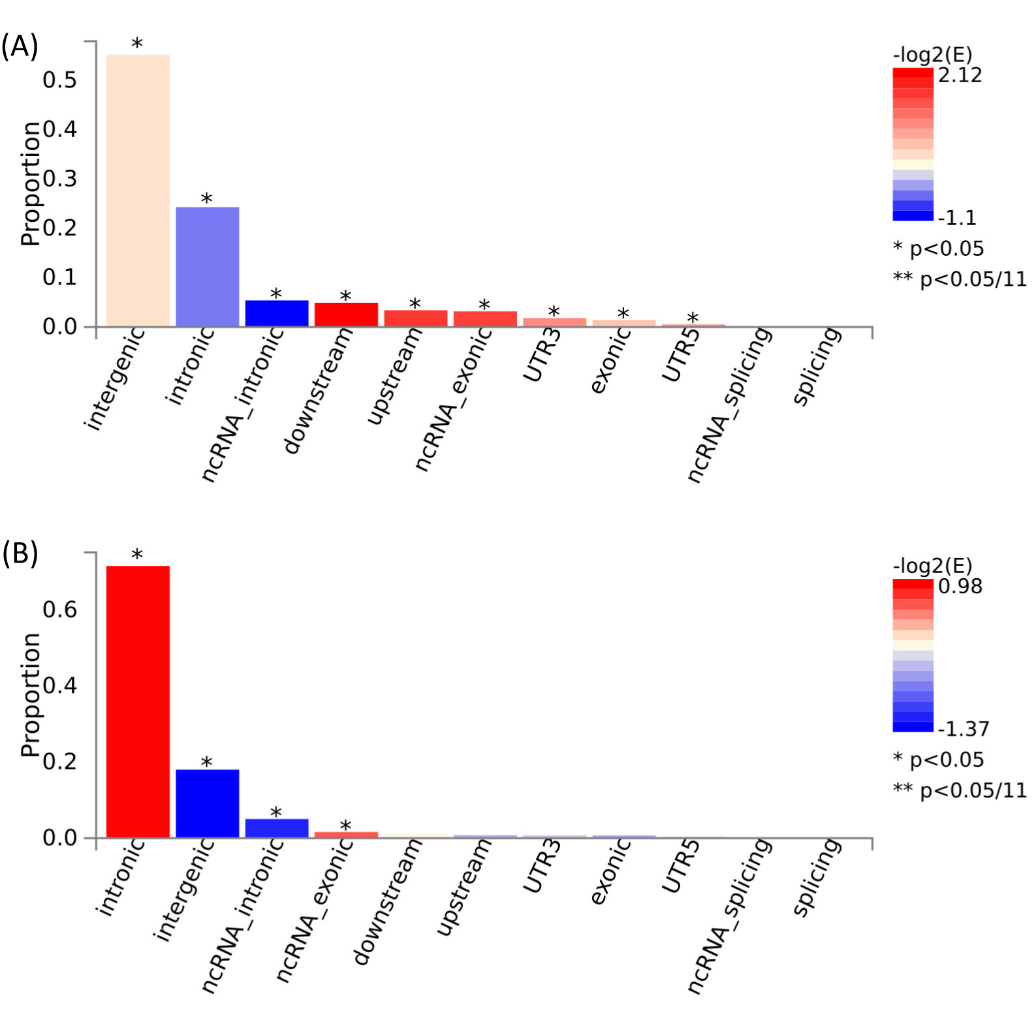


**Figure S7.** Functional effects of pleiotropic SNPs on genes. (A) MDD and FT4 normal; (B) MDD and TSH total.


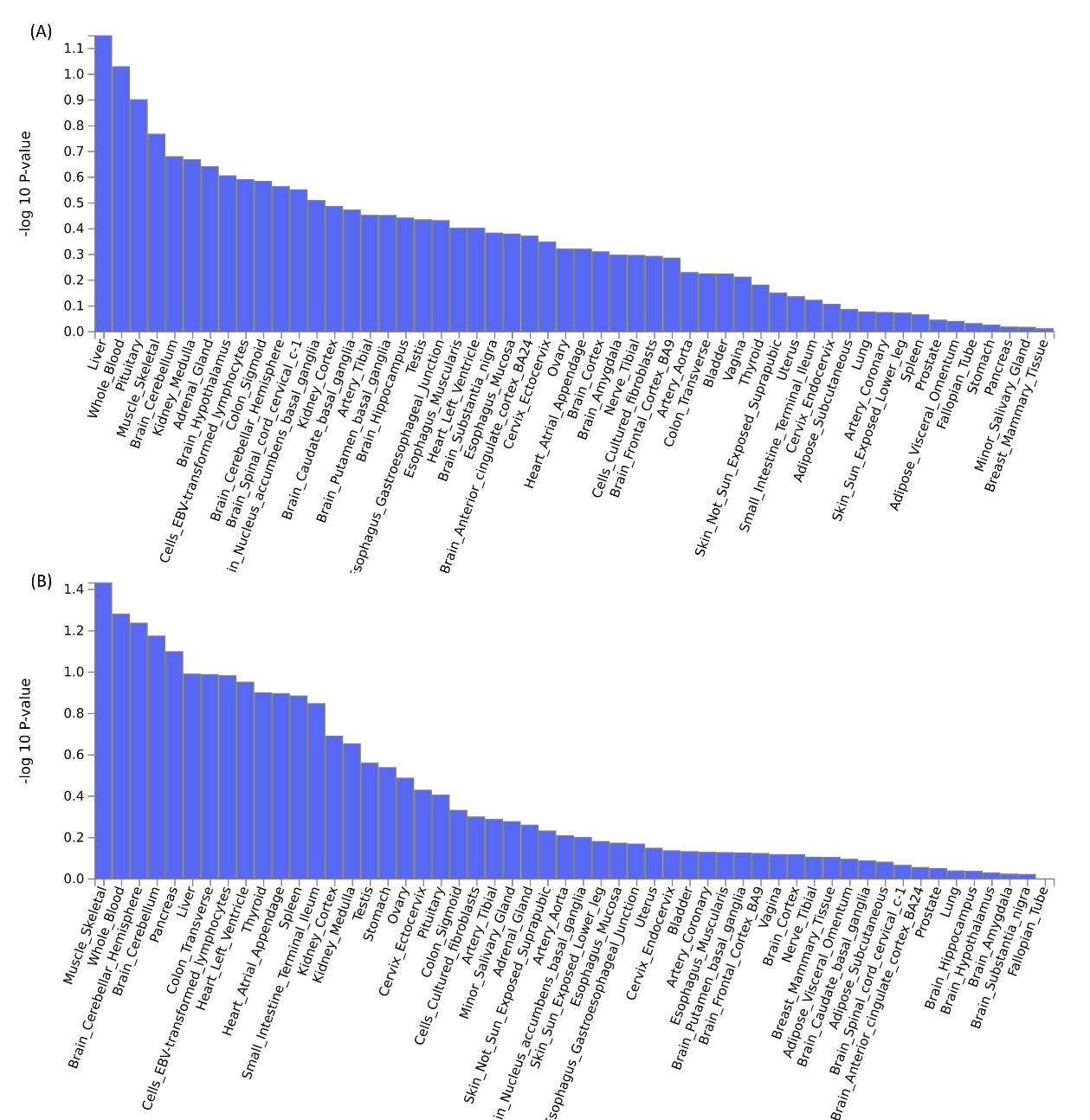


**Figure S8.** Enrichment analysis of pleiotropic effects in different tissues. (A) MDD and FT4 normal; (B) MDD and TSH total.


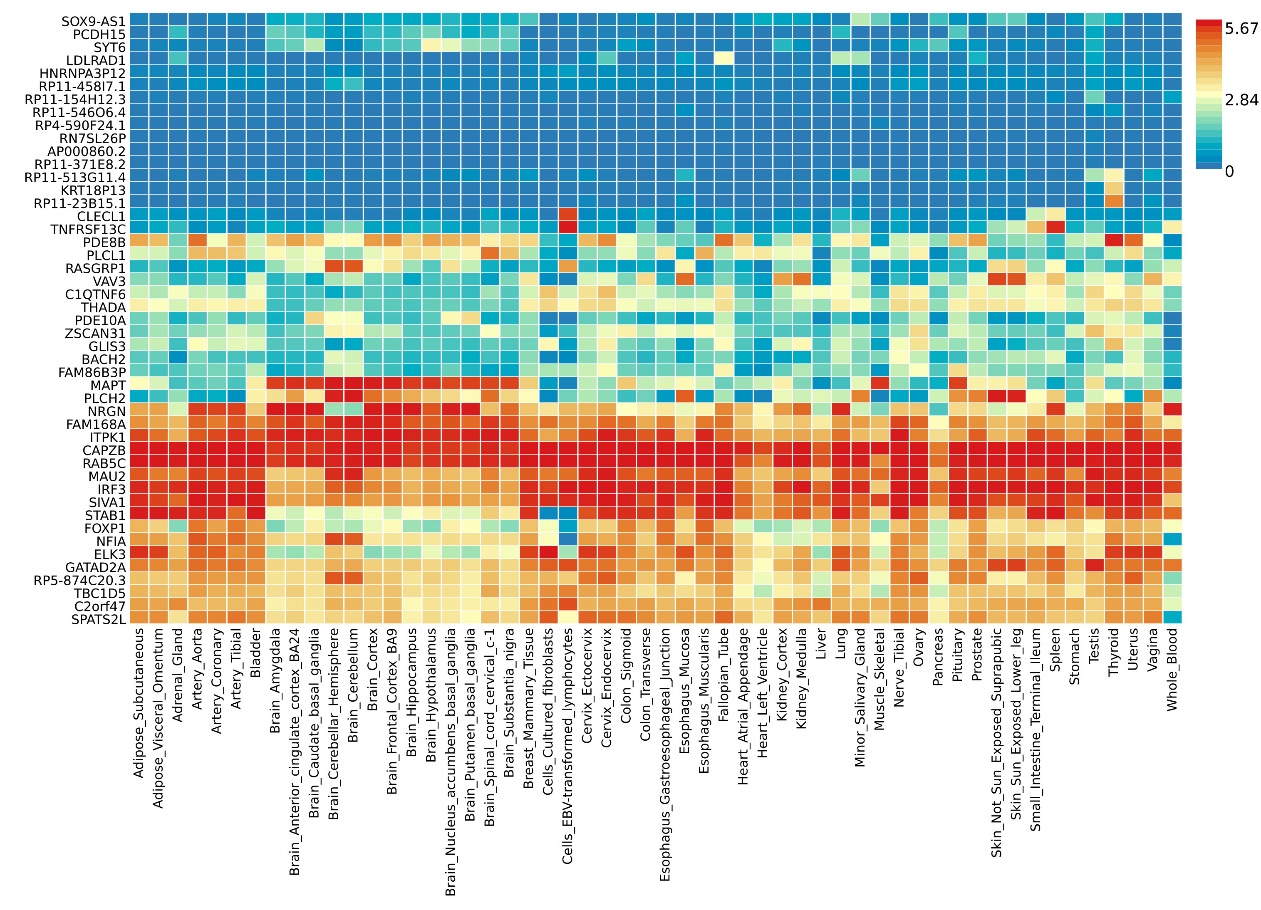


**Figure S9.** Expression of genes matched by pleiotropic loci in different tissues. Red represents high expression and blue represents low expression; the horizontal coordinates are the 54 GTEx tissues and the vertical coordinates are the identified pleiotropic genes.


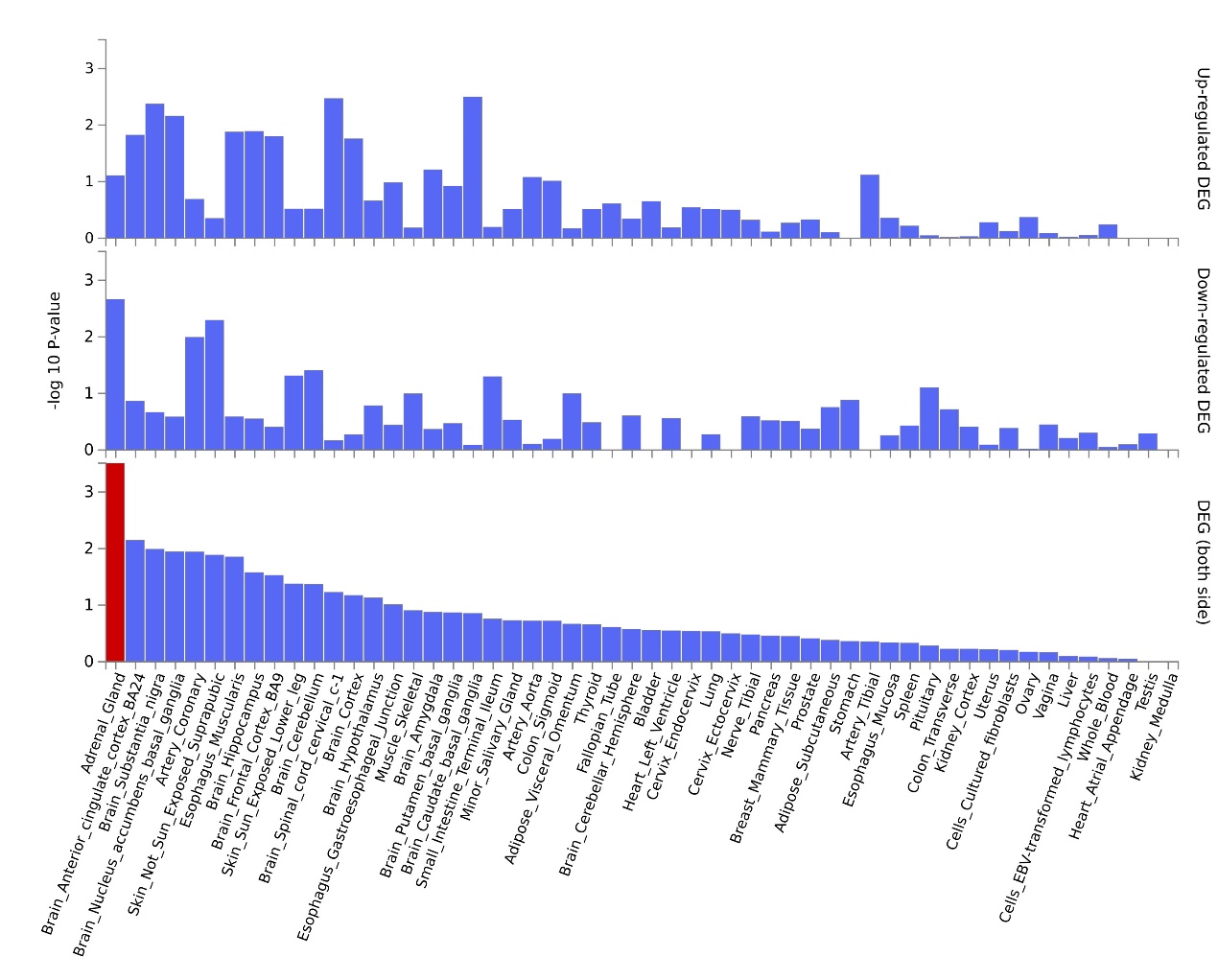


**Figure S10.** Enrichment of genes matched by pleiotropic loci in different tissues. Ordered by significance from left to right, tissues with significant multiple corrections are shown in red. Horizontal coordinates indicate the 54 GTEx tissues and vertical coordinates indicate the *P*-values.


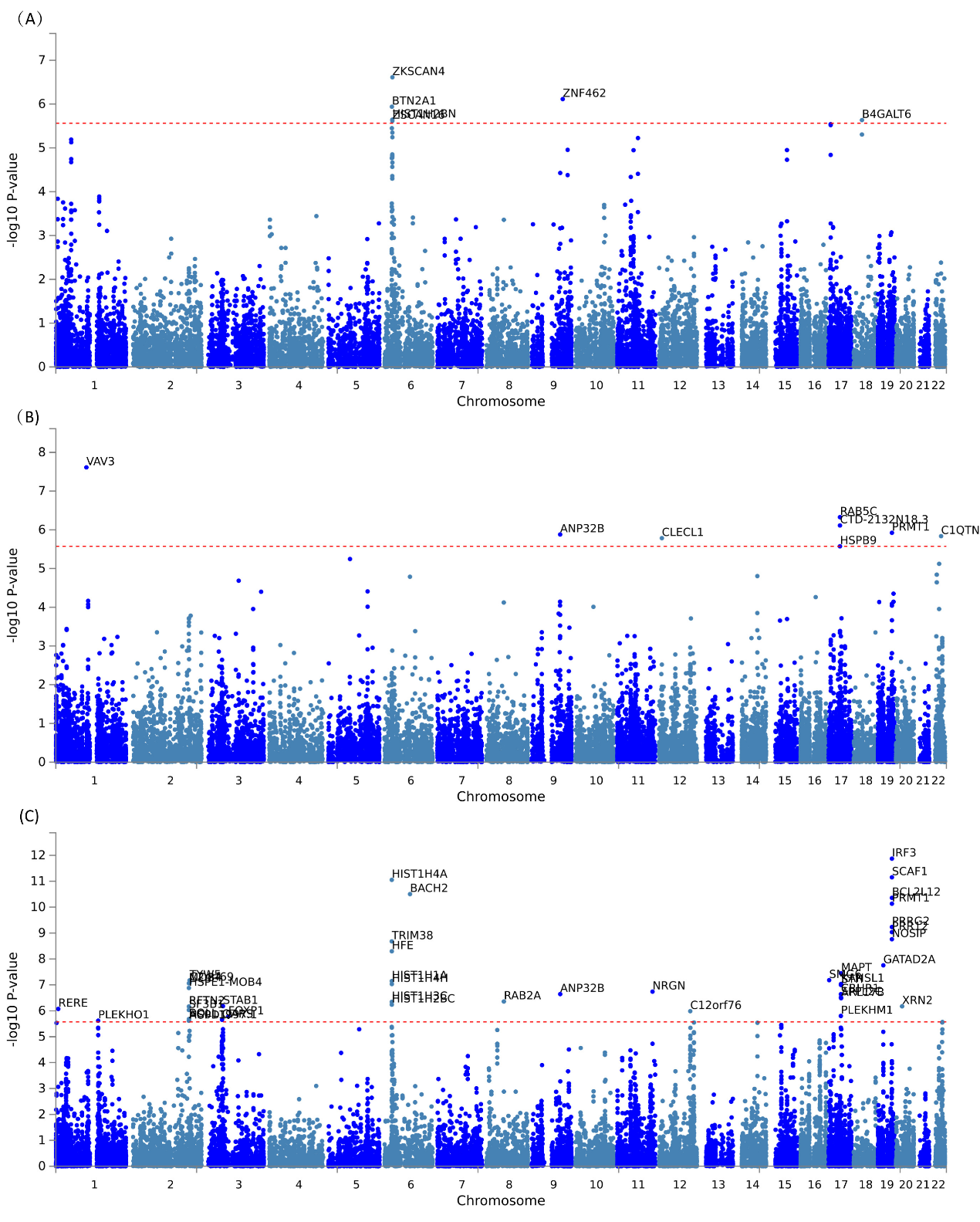


**Figure S11.** Manhattan plots of MAGMA gene analysis. (A) Hypothyroidism and MDD; (B) Hypothyroidism and ANX; (C) Hypothyroidism and SCZ. The red line represents the significance level of multiple corrections.


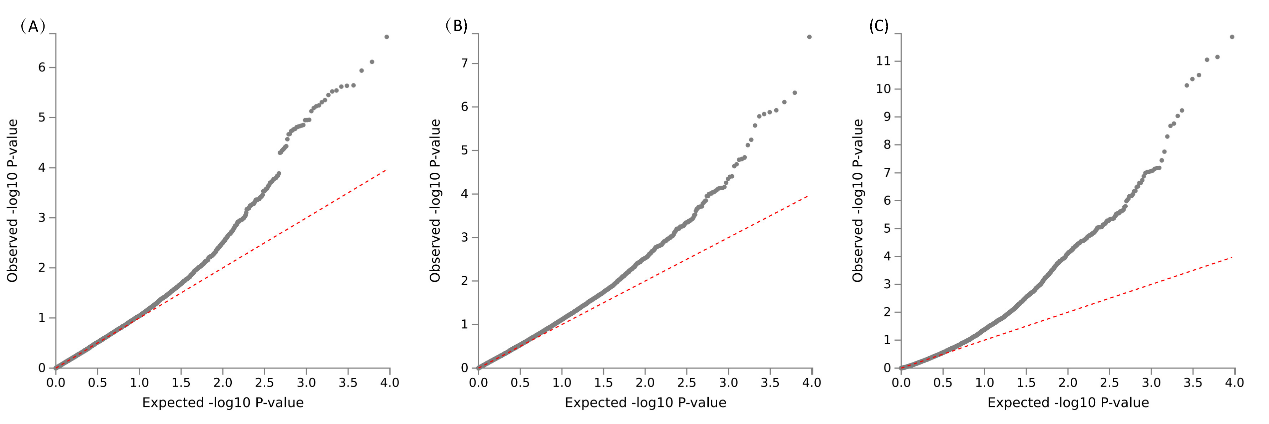


**Figure S12.** QQ plots of MAGMA gene analysis. (A) Hypothyroidism and MDD; (B) Hypothyroidism and ANX; (C) Hypothyroidism and SCZ.


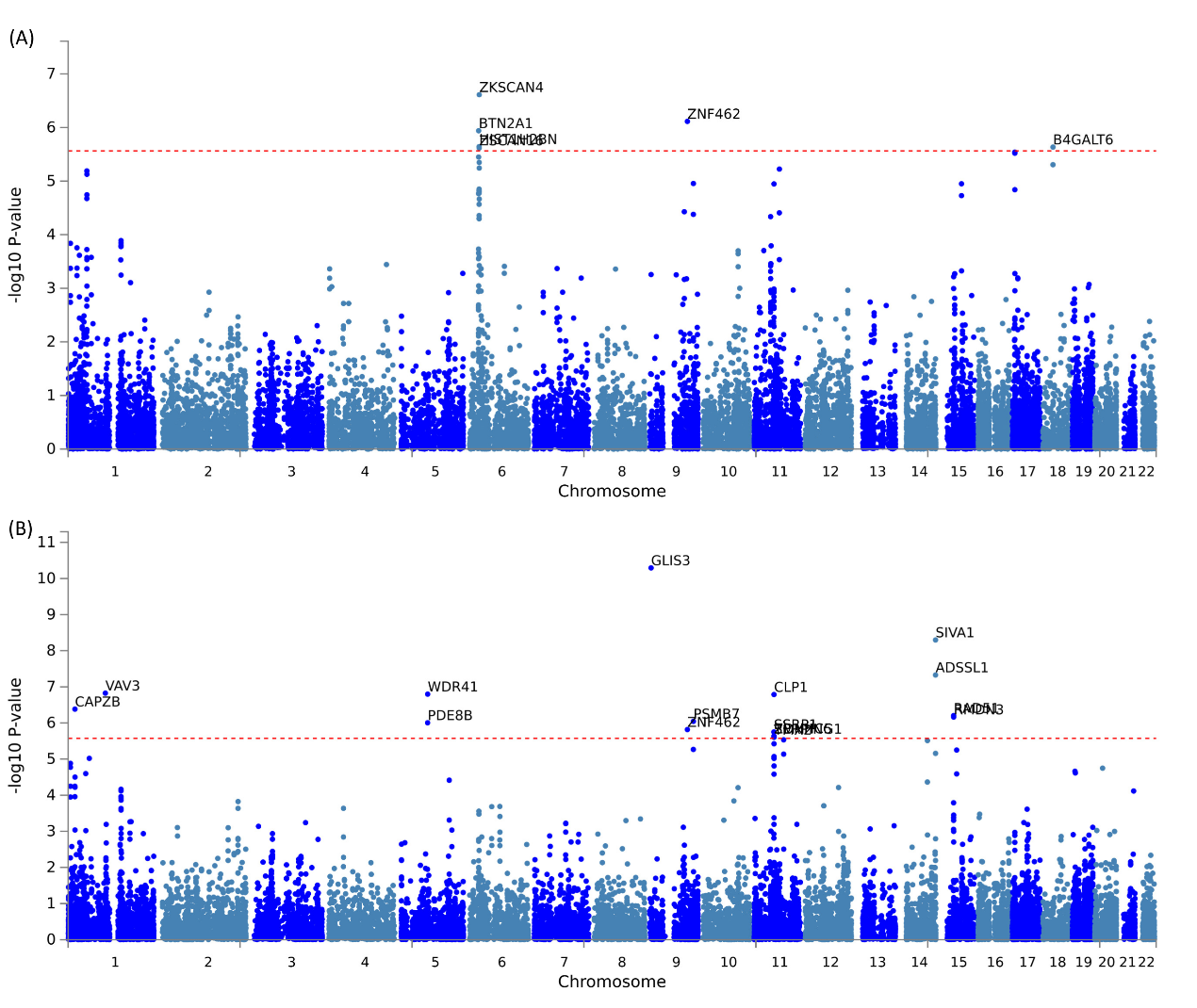


**Figure S13.** Manhattan plots of MAGMA gene analysis. (A) MDD and FT4 normal; (B) MDD and TSH total.


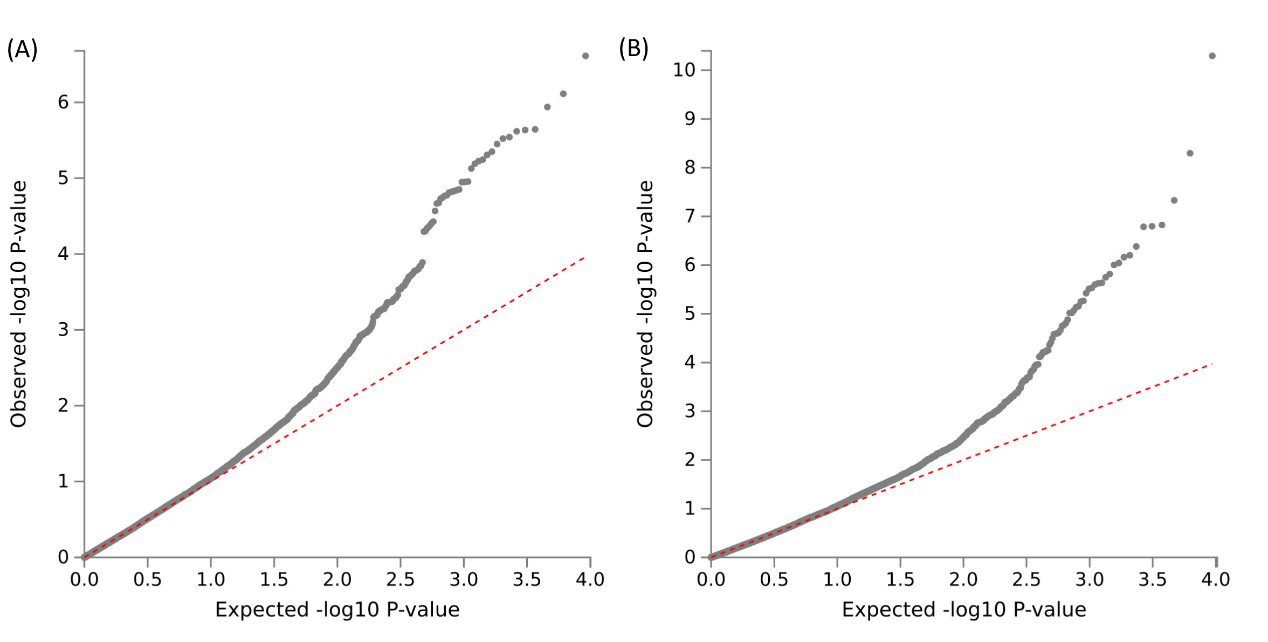


**Figure S14.** QQ plots of MAGMA gene analysis. (A) MDD and FT4 normal; (B) MDD and TSH total.


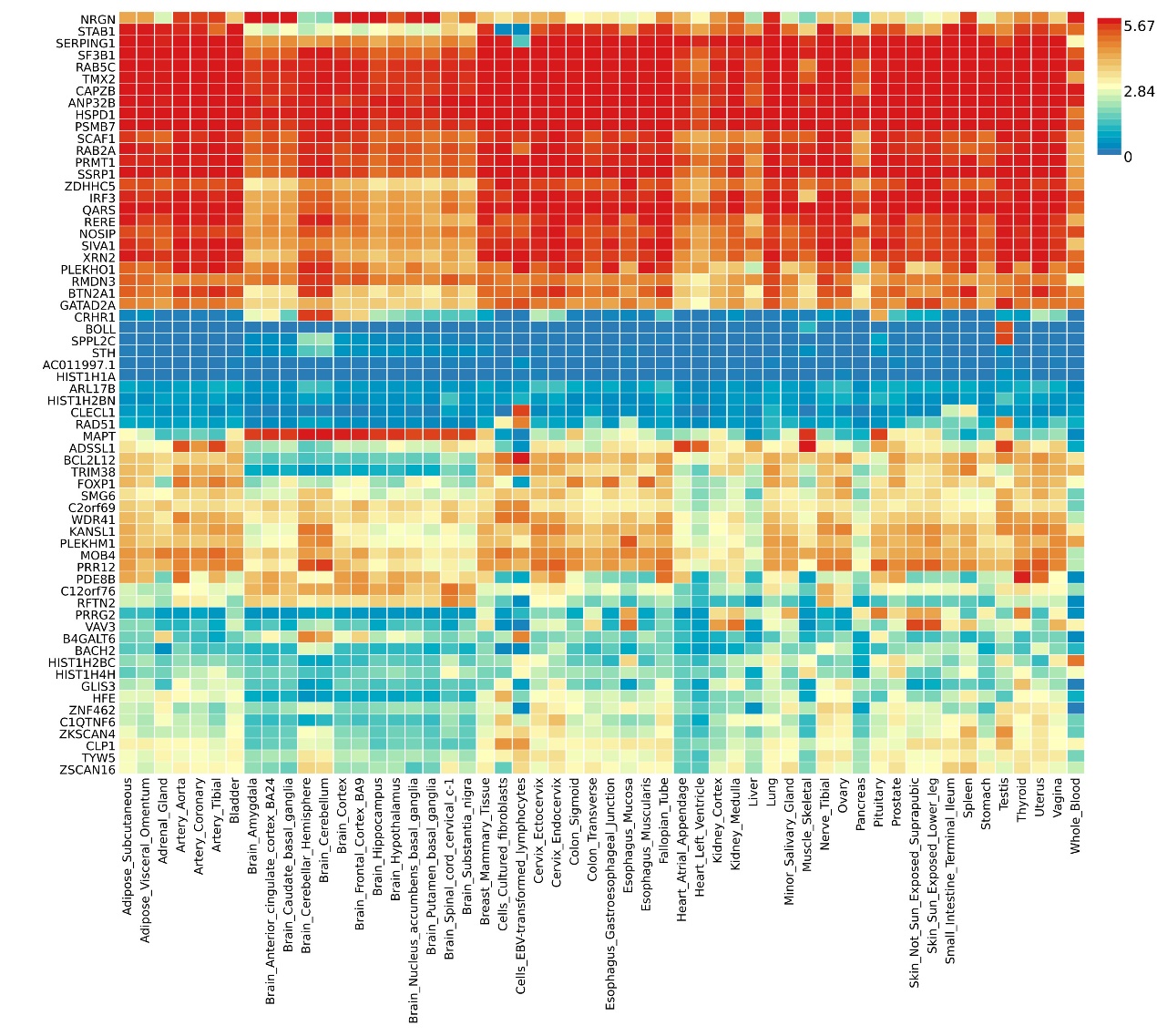


**Figure S15.** Expression of pleiotropic genes in different tissues.


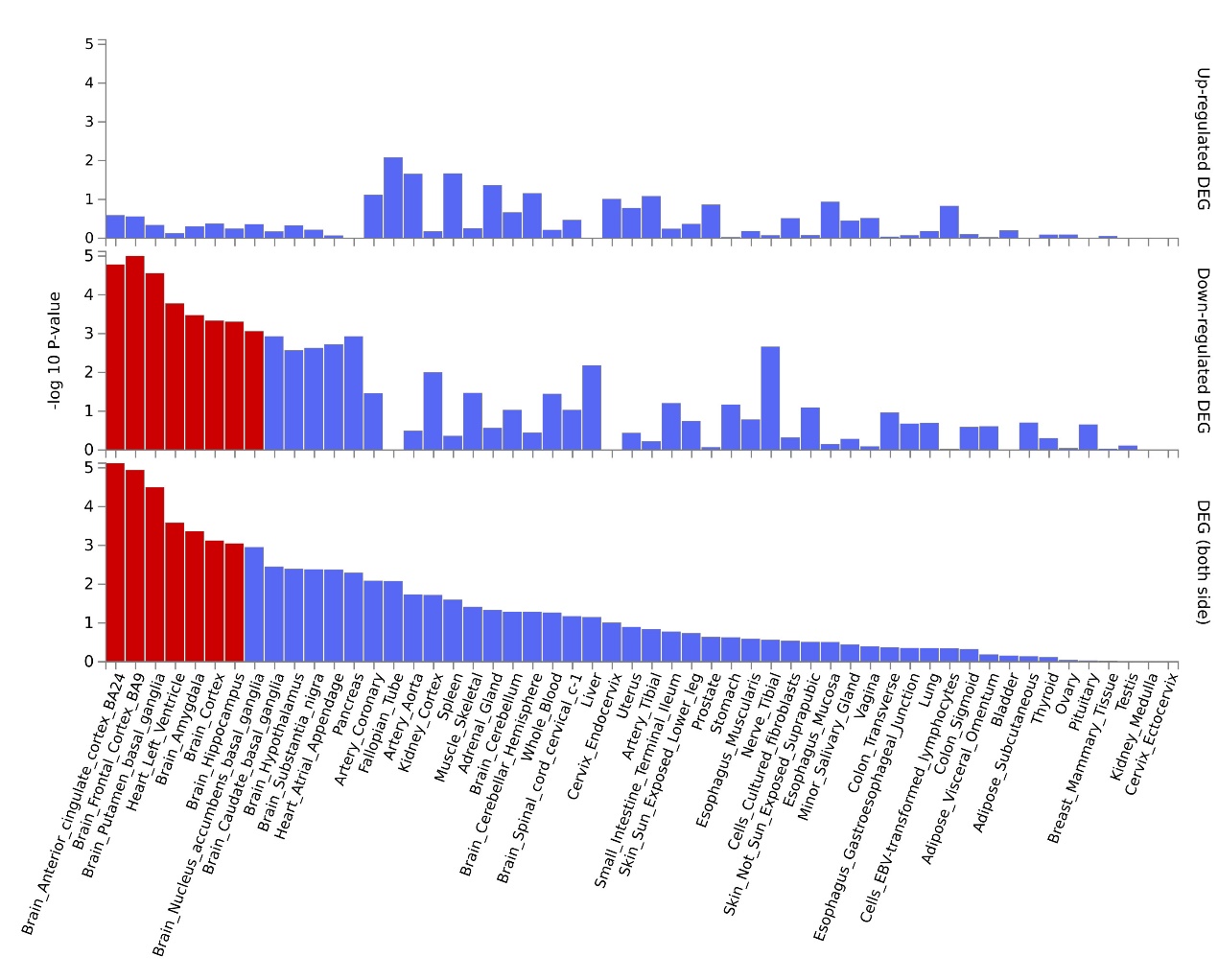


**Figure S16.** Pathway enrichment of pleiotropic genes.


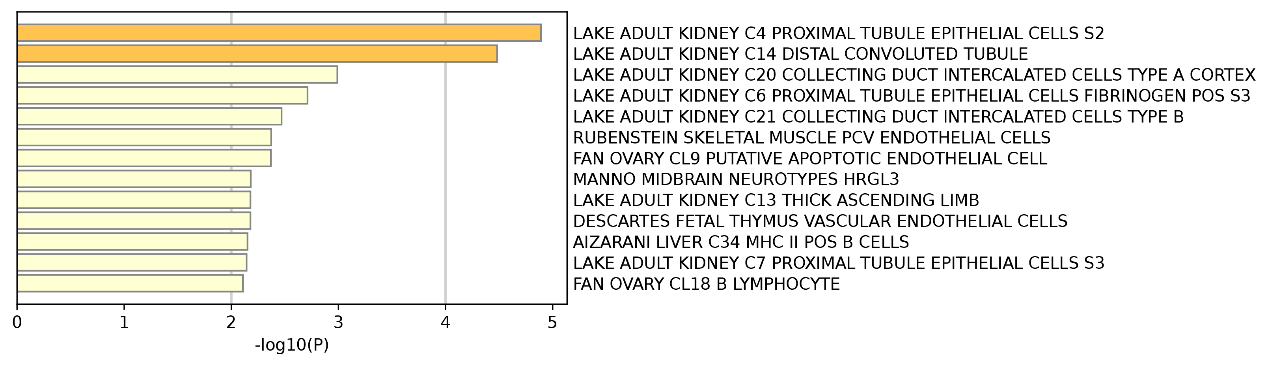


**Figure S17.** Results of cell type enrichment analysis of pleiotropic genes.


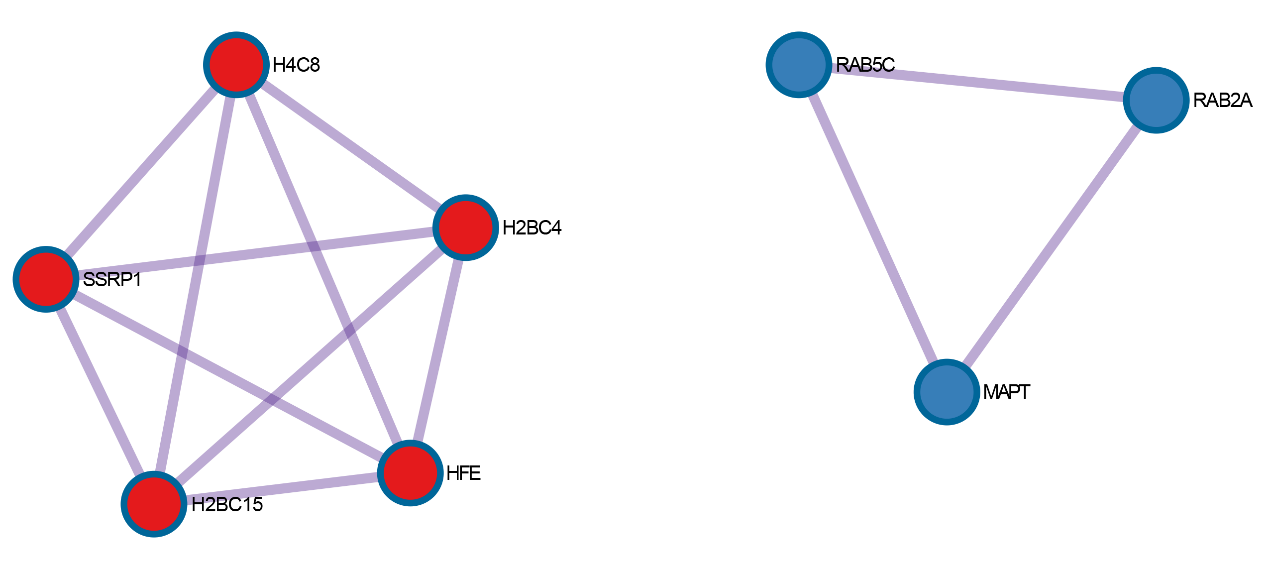


**Figure S18.** Results of protein interaction network analysis.
